# Quantifying word informativeness and its impact on eye-movement reading behavior: Cross-linguistic variability and individual differences

**DOI:** 10.3758/s13428-025-02878-x

**Published:** 2025-11-11

**Authors:** Inbal Kimchi, Sascha Schroeder, Noam Siegelman

**Affiliations:** 1 Department of Psychology, The Hebrew University of Jerusalem, Mount Scopus Campus, 9190501 Jerusalem, Israel; 2 Institute of Psychology, University of Goettingen, Göttingen, Germany

**Keywords:** Reading, Eye movements, Cross-linguistic differences, Individual differences

## Abstract

The importance or centrality of a linguistic unit to a larger unit’s meaning is known to affect reading behavior. However, there is an ongoing debate on how to quantify a unit’s degree of importance or centrality, with previous quantifications using either subjective ratings or computational solutions with limited interpretability. Here we introduce a novel measure, which we term “informativeness”, to assess the significance of a word to the meaning of the sentence in which it appears. Our measure is based on the comparison of vectorial representations of the full sentence with a revised sentence without the target word, resulting in an easily interpretable and objective quantification. We show that our new measure correlates in expected ways with other psycholinguistic variables (e.g., frequency, length, predictability), and, importantly, uniquely predicts eye-movement reading behavior in large-scale datasets of first (L1) and second language (L2) readers (from the Multilingual Eye-tracking Corpus, MECO). We also show that the effects of informativeness generalize to diverse writing systems, and are stronger for poorer than better readers. Together, our work provides new avenues for investigating informativeness effects, towards a deeper understanding of the way it impacts reading behavior.

**Supplementary Information:**

The online version contains supplementary material available at 10.3758/s13428-025-02878-x.

## Introduction

Our ability to read is a highly complex behavior that requires mastery of multiple sub-skills. These include, among others, the ability to parse the orthographic (i.e., written) input, convert written symbols to sound and meaning, move the eyes efficiently across the text, and integrate incoming meaning with already-read material. In recent years, our theoretical understanding of these various sub-processes has been advanced substantially by *Statistical Learning views of reading*, which emphasize the fundamental importance of assimilating statistical regularities within the writing system at various levels of the input for achieving proficient reading skills. According to this view, as children acquire literacy skills, they gradually develop sensitivity to various patterns present in their written language. Then, at the end of this developmental trajectory, proficient readers utilize these statistical regularities to read more efficiently (e.g., Arciuli, 2018; Frost, 2012; Sawi & Rueckl, 2019). Furthermore, per this view, individual differences in reading can be tied to variation in readers’ ability to learn and utilize different regularities in their writing system (e.g., Rueckl, 2016; Siegelman et al., 2020), and cross-linguistic differences may be attributed to differences in the availability and reliability of different regularities across writing systems (e.g., Seidenberg, 2011; Treiman & Kessler, 2022).

To understand the full scope of reading ability as well as individual- and cross-linguistic variation, a major goal of statistical learning views of reading has been to precisely quantify the various cues and regularities that exist in writing systems and examine how they impact reading behavior. Historically, a large portion of this work has been inspired by The Triangle Model of Reading, which posits that learning to read involves forming associations between words’ orthography, phonology, and semantics (Plaut et al., 1996; Seidenberg & McClelland, 1989). Indeed, substantial work has gone into the quantification of the regularities between the “nodes” of the triangle, focusing on orthographic-phonological (O-P) and orthographic-semantic (O-S) associations, showing that they influence visual word recognition performance (e.g., Baron & Strawson, 1976; Ehri, 2005; Jared et al., 1990; Rastle et al., 2000; Seidenberg & Gonnerman, 2000; Amenta et al., 2023; Marelli et al., 2015). Studies have also shown that individual differences in reading development can be attributed to variation in sensitivity to O-P and O-S (Siegelman et al., 2020; Siegelman, Rueckl, et al., 2022a, b). This evidence jointly emphasizes the foundational role of word-level regularities – that is, the intrinsic characteristics of individual words (i.e., how letters and letter chunks map onto spoken forms and meaning) – in shaping reading behavior.

## Cues in print beyond the level of single words

Importantly, however, regularities in the O-P and O-S mappings encompass only a small part of a presumably broader array of cues and regularities that are available in print and can be utilized by readers. In particular, such word-level regularities do not cover additional available sources of information that have to do with the relations *between words in sentences and longer texts* (nor the relation between larger units such as multiword units or phrases), which proficient readers utilize to enhance their efficiency when reading sentences and longer passages (and see Amenta et al., 2023 for evidence of interactions between word- and text-level regularities).

Indeed, alongside work on O-P and O-S regularities, which was done primarily within the single-word recognition research tradition, researchers in the eye-movement research community have long been interested in specific types of word-to-sentence (or text) relations. Arguably, the most prominent example is work on predictability effects: The predictability of an incoming word given its preceding text (see Staub, 2015, for review). Indeed, numerous attempts have been made to precisely quantify the extent of words’ predictability in a given context, utilizing diverse methods, from classic Cloze Predictability tasks (dating back to Taylor, 1953; see also, e.g., Lowder et al., 2018; Rayner & Well, 1996) to contemporary Large Language Models (e.g., Berzak & Levy, 2023; Cevoli et al., 2022; Hofmann et al., 2022; Kun et al., 2023). Historically, work on word predictability effects has often utilized artificial sentences where a target word is embedded in a highly predictable or unpredictable context (e.g., Kliegl et al., 2004; Rayner et al., 2011), but more recently, the continuous impact of the extent of a word predictability has been demonstrated also in naturalistic text reading (e.g., Cevoli et al., 2022; Wilcox et al., 2023).

The importance of word predictability to reading behavior notwithstanding, it is also evident that the predictability of an individual word *N* based on its preceding context up to word *N-1* does not constitute the sole wellspring of information encompassed in texts. Our focus here is on an additional feature that readers presumably consider and utilize during text reading – *the importance of a word to the meaning of the full sentence in which it appears*. With this goal in mind, in the current paper, we present a novel measure for the degree of the *informativeness* of a word within a sentence, utilizing developments in large language models.

## Informativeness and centrality: The common approach

Our *informativeness* measure (presented in detail below) is conceptually related to a previously explored construct, often referred to as *centrality*. Typically, work on centrality aimed at identifying how central a part of a text is (e.g., a given sentence within a text; or a more abstract idea or event described in a text), to the overall message or content of a text. Operationally, earlier research has typically evaluated centrality using ratings from readers. In this approach, readers are asked to rate the importance of each idea in relation to the overall meaning of the passage, for example, using a Likert scale (Miller & Keenan, 2009; Miller et al., 2012; Meyer & McConkie, 1973; Lorch et al., 1999; Johnston & Afflerbach, 1982; Yeari et al., 2015; Jayes et al., 2022; Dirix et al., 2020). Related approaches utilized causal network analysis, which also relies on human judgements, and have been found to predict judgments of importance, with the importance rating of a statement increasing proportionally with the number of direct causal connections and to whether it was a part of a causal chain (Trabasso & Sperry, 1985). Other approaches employed different experimental manipulations to alter the presumed centrality of elements to examine their impact on reading behavior, for example, by manipulating the frequency of a concept’s occurrence within a passage (Albrecht & O’Brien, 1991), or by experimentally altering readers’ goals (e.g., Kaakinen et al., 2002).

Regardless of the exact measure of centrality used, research has shown that after reading a text, recall increases as a function of centrality of an idea or a text segment: That is, the more central an idea is to the overall meaning of the text, the more likely it is to be recalled quickly and accurately (Miller & Keenan, 2009; Miller et al., 2012; Miller & Keenan, 2011; Yeari et al., 2015; Albrecht & O’Brien, 1991; Lorch et al., 1999; Kaakinen et al., 2002). Furthermore, individuals with reading difficulties, including those with attention deficit hyperactivity disorder (ADHD), poor readers, or non-native language readers, have been shown to exhibit a weaker centrality effect, leading to what was labeled as a “centrality deficit” (Miller & Keenan, 2009; Miller et al., 2012; Miller & Keenan, 2011).

Studies that tracked eye movements during text reading have provided further insights into centrality effects and their deficits in individuals with reading difficulties. In addition to again showing that these groups tend to recall fewer central ideas compared to control participants in offline recall tasks, eye-movement studies showed that all groups, including individuals with ADHD or poor comprehension skills, exhibited longer fixation durations and higher re-reading rates for central ideas than peripheral ones (Jayes et al., 2022; Yeari et al., 2018; Lev, 2020; Schifer, 2019; although these effects may vary across reading tasks; see Yeari et al., 2015). However, centrality effects were not observed in all studies, which have been attributed to the subjectivity of the measure, as evident in the variations in the definition of units and raters’ expertise (see Dirix et al 2020). Further, studies have also shown that poorer readers (e.g., individuals with ADHD or poor comprehension skills) were found to spend *increased effort* (e.g., longer fixation times, more rereading) on central ideas compared to control groups (although there is variation across studies in the exact eye-movement measures showing this variation). This suggests that these individuals may employ compensatory strategies, such as dedicating more time to rereading central ideas, in an attempt to cope with their reading difficulties (see Yeari et al., 2018; Lev, 2020; Schifer, 2019; but see Yeari & Lev, 2021, for counterevidence).

## Towards a computational approach to quantifying informativeness

A limitation in studies that use manually coded centrality or importance measures lies in their subjectivity, making it challenging to scale the analysis consistently and objectively across datasets or studies, as well as to different languages. The inherent subjectivity underscores the necessity for more scalable and objective approaches. Indeed, in parallel to the main line of centrality research reviewed above, computational approaches have recently emerged to assess the centrality or relative importance of different text segments within larger texts and examine their impact on eye-movement reading behavior. Such measures have been made possible due to recent advances in distributional semantic models (DSM), a wide set of approaches that provide vectorial numeric representations of the meaning of different text segments (words, multiword units, and sentences), and can thus be used to examine the semantic relations between various parts of texts (see Günther et al., 2019 for review). Indeed, DSM-based measures have been used extensively in literature on word predictability, generally, by quantifying the similarity between the vectorial embedding of an incoming word and the embedding of its preceding context (e.g., Hofmann et al., 2022; Pynte et al., 2008; Salicchi et al., 2021). In what follows, we review previous quantifications of centrality or informativeness based on vectorial embeddings, which then leads us to our current operationalization.

A recent study by Fan and Reilly (2020), for example, investigated the impact of informativeness defined via embedding similarities on durational eye-movement measures during passage reading, in Chinese elementary school students in two longitudinal data points (grades 4 and 5). As measures of informativeness, Fan and Reilly examined the semantic similarity between smaller units of text and larger units in which they appeared. These included a measure of word-sentence similarity, assessing the semantic similarity between the embeddings representing each word and its containing sentence; and a measure of sentence-paragraph similarity, which quantified the semantic similarity between each sentence and its containing paragraph. Given their focus on Chinese, where orthographic words are unspaced, words were defined based on segmentation algorithms (with dictionary lookups in case of ambiguities) and spanned between 1 and 5 characters. Similarity was defined as the cosine of the angle between the two relevant units of texts (embeddings extracted from Google’s Sentence Encoder model). The results of the analyses by Fan and Reilly, however, produced little evidence for stable effects of informativeness on eye-movement patterns in children. This might be due to their focus on developing readers (who may still not be sensitive to this high-order cue), or limitations of their operationalization of informativeness. In particular, a potential issue with their approach is that the similarity between a word and the full sentence it is embedded in does not reflect informativeness specifically. Thus, a word’s meaning may not be semantically related to the overall meaning of the sentence, yet still crucial to the intended message (e.g., the word “wedding” is not similar in semantic space to the sentence “the wedding was ruined by the heavy rain and snow”, yet it is crucial for the intended meaning). Similarly, a word can be closely related to the sentence’s meaning but not particularly informative (in “the royal couple married in a beautiful wedding ceremony”, the meaning of “wedding” strongly relates to the sentence’s meaning, but that word does not add much new information and is not particularly important for the message). Our current operationalization therefore offers a new and more targeted measure of informativeness.

Other recent attempts to quantify informativeness utilized the Transformer architecture of large language models (Vaswani et al., 2017). Two such measures in particular have been recently used. The first is the *attention weights* allocated to different words in a given text, which is a key component in Transformer architecture. Recent studies have claimed that the internal attention weights of a Transformer model can provide a measure of a word’s importance in a given text, and indeed showed that attention weights predict eye-movement reading measures (e.g., Bensemann et al., 2022; Sood et al., 2020). A second measure extracted from Transformer models is *saliency*, which is a model’s gradient of the output corresponding to the correct prediction with respect to parts of the input. It has been argued that saliency can be used to identify parts of the input that have the biggest influence on the prediction (i.e., representation) of a larger text unit, and, therefore, their relative importance. Indeed, recent studies again tied saliency to various eye-movement reading measures (e.g., Hollenstein et al., 2021; Hollenstein & Beinborn, 2021; Morger et al., 2022). While these studies, collectively, offer intriguing computational ways to quantify a word’s informativeness, we argue that their limitation is in their lack of interpretability and transparency. Large Language Transformer models are highly complex, and the extent to which theoretical psycholinguistic concepts (e.g., “informativeness” or “centrality”) can be tied to specific computational features internal to these models (e.g., “attention weights”, “saliency”) is an open question (the complexity of this issue is also apparent in the non-trivial links between different measures presumably tied to informativeness, see Jain & Wallace, 2019; Serrano and Smith, 2019). In the current work, we therefore opted for a more transparent definition of a word’s informativeness: That is, we still utilize state-of-the-art LLMs that produce high-quality representations of words and sentences, but avoid relying on measures internal to the models and their architecture.

## The current study: A new measure of words’ informativeness

Building upon these recent computational attempts, the goal of the current work is to take a step forward by proposing a new interpretable yet objective measure of a word’s informativeness in relation to the meaning of the sentence in which it appears. In a nutshell, the new informativeness measure is based on a comparison of the vectorial embedding of the full containing sentence and the embedding of a revised sentence *without the word*. This measure is grounded in the notion that omitting a word that is more informative to the full sentence’s meaning should result in a more dissimilar meaning of the revised sentence, reflecting higher informativeness (see details below). In contrast, omitting a word of little importance should not drastically alter the meaning of the sentence, reflecting lower informativeness. To reiterate, our approach (1) avoids subjective decisions, much like other computational approaches; (2) is easily interpretable; and (3) can be easily used in different languages (by utilizing recent multilingual sentence embeddings). Furthermore, although the current paper focuses on the informativeness of individual words within sentences, our computational approach is extendable to smaller-to-larger segments of text (see more in the General Discussion).

In what follows, we start by presenting our measure, and the available datasets of eye movements during reading that we use for its validation (from the Multi-lingual Eye-movement Corpus, MECO; Kuperman et al., 2023; Siegelman, Schroeder, et al., 2022). We also provide descriptive statistics on how the informativeness of words correlates with basic psycholinguistic measures (length, frequency, predictability) and varies across languages. Then, we turn to the results of our validation analyses, in which we test the novel measure against behavioral eye-movement data to investigate the effects of word informativeness on eye-movement reading behavior in both first (L1) and second (L2) language readers. To preview our findings, our results show that (1) informativeness has an impact on multiple eye-movement measure, with particular notable effects on later eye-movement measures (i.e., total reading time, re-reading); (2) the effects of informativeness span different languages (although some interactions with writing system do emerge); and (3) reading skill levels interacts with word informativeness, with poorer readers more strongly impacted by informativeness in their online reading behavior. These findings, which are generally in line with previous reported findings and our hypotheses, provide support for the utility of our quantification approach. At the same time, they also highlight some refinements to existing studies and highlight open questions that future research could tackle with the new quantification offered.

## Methods

### Participants, materials, and procedure

For this study, we utilized data from the Multilingual Eye-tracking Corpus (MECO), an international collaborative project that provides cross-linguistic eye-tracking data for reading research (Siegelman, Schroeder, et al., 2022; Kuperman et al., 2023). The first Wave of MECO encompasses eye-movement passage reading data from 13 different testing sites in 13 countries.

There are two main components to the MECO corpus, labeled MECO-L1 and MECO-L2. The first is a collection of participants’ eye movements in their native language. This dataset was collected by presenting participants with a set of 12 texts in their first language (L1). Among these texts, five were translations from English to the participants’ respective L1s (i.e., translation-equivalents across all tested languages), while the remaining seven texts were non-matched texts on similar topics, created by each research team in the target language. The second component of the corpus, MECO-L2, includes an additional eye-tracking reading task in English, which was the second language (L2) for participants in 11 out of the 12 testing sites (with the exception of the Canadian, English L1, sample; one site opted out of participating in the MECO L2 part of the study, and therefore MECO L2 has one site less overall than MECO L1). In addition to the central eye-tracking reading tasks, MECO participants completed a range of individual differences tests in both their L1 and their L2. Below we use data from the battery of L2 (i.e., English) individual differences tests, which included tests of timed word and pseudoword naming (Test of Word Reading Efficiency, TOWRE, Torgesen et al., 2012), lexical decision (Lexical Test for Advanced Learners of English, LexTALE, Lemhöfer & Broersma, 2012), vocabulary knowledge (adapted from Nation & Beglar, 2007), and spelling (adapted from Andrews & Hersch, 2010; for more details regarding these individual differences tests, see Kuperman et al., 2023). The inclusion of these measures allows us to explore how the effects of informativeness on eye movements vary as a function of individual differences in English reading skill.

The datasets we utilize below include a total of *N* = 580 participants in analyses of MECO-L1, and *N* = 543 participants in MECO-L2. The breakdown of participants to testing sites (and MECO-L1 languages) is available in the original MECO papers: See Siegelman, Schroeder, et al., 2022 and Kuperman et al., 2023. We excluded outliers at the interest-area (word token) level from both datasets by removing observations with total fixation times shorter than 80 ms or longer than 2000 ms, as well as interest areas with first fixation duration longer than 800 ms or gaze duration longer than 1000 ms (all these values presumably reflect unrealistic fixation times, related to tracker failure or other data issues). This resulted in filtering ~3% of interest areas in both MECO-L1 and MECO-L2. We used frequency counts from the multilingual Open Subtitles Corpus (Lison & Tiedemann, 2016) for MECO-L1 and from the English Lexicon Project (Balota et al., 2007) for MECO-L2. We also quantified the predictability of words in context, as an additional covariate in our models, to ensure that the effects observed for informativeness are not merely due to predictability (see below). Predictability was defined as the cosine similarity between the vector representation of the specific word and the vector representation of the part of the sentence preceding it. Following a recent study (Bianchi et al., 2020), we used the multilingual fastText word embeddings for the quantification of word predictability (Bojanowski, Grave, et al., 2017)^1^.

### The informativeness measure

We propose a novel metric, henceforth labeled as a word’s “informativeness”, to quantify the significance or importance of a word to the meaning of the sentence in which it appears. Our measure leverages an extension of the model BERT (Bidirectional Encoder Representations from Transformers), called sentence-BERT (Reimers & Gurevych, 2019), which is designed to provide vectorial semantic representations of full written sentences. Sentence-BERT is particularly useful for estimating the informativeness of words within sentences, because (1) unlike some previous DSMs, it provides sentence-level representations that are sensitive to word order and other sentence-level properties, and (2) it can produce vectorial representations of sentences in various languages, which is useful for our analyses of the MECO-L1 multilingual data. In the work below we use pre-trained versions of sentence-BERT from the Python framework *sentence-transformer* (Reimers & Gurevych, 2019). To estimate informativeness in English as well as other languages, we use the multilingual pre-trained model *distiluse-base-multilingual-cased-v2v* (Reimers & Gurevych, 2019, 2020). Note that in this work, we focus on quantifying the informativeness of printed orthographic words. We focus on the orthographic word level because our work here deals with alphabetic writing systems where words are marked with spaces, and because the eye-movement MECO data used for validation includes dependent variables at the word-token level (but see General discussion for a discussion of the implications of this decision, and future extensions and changes to the basic unit of analysis).

To calculate the informativeness of a particular word in a sentence, we consider the vectorial representation of the entire sentence as well as the vectorial representation of a revised sentence *with the exclusion of that word*. Then, a potential measure of the informativeness of word *i* within sentence *j* is:

1 $${Informativeness}_{\left(word-i, sentence-j\right)}=1-\text{cosine }(sentence-j, sentence-j-w/o-word-i)$$

Under this definition, values approaching 1 indicate that a word is important to the meaning of the sentence: That is, the meaning of the full sentence is orthogonal to the meaning of the sentence without that word. In contrast, values approaching 0 mean that the word is not important to the meaning of the sentence (the meaning remains highly similar with or without the word)^2^.

However, omitting most words in natural sentences results in very minor changes in meaning, and thus the raw difference value in Eq. (1) results in a skewed measure with the vast majority of values close to 0. Hence, to reduce the skewness of the measure, we apply a natural-log transformation to the values in formula (1). That is, the measure we use in our analyses below is defined as:

2 $${Informativeness}_{\left(word-i, sentence-j\right)}=\text{In }\left(1-\text{cosine }(sentence-j, sentence-j-w/o-word-i)\right)$$

**Fig. 1:**
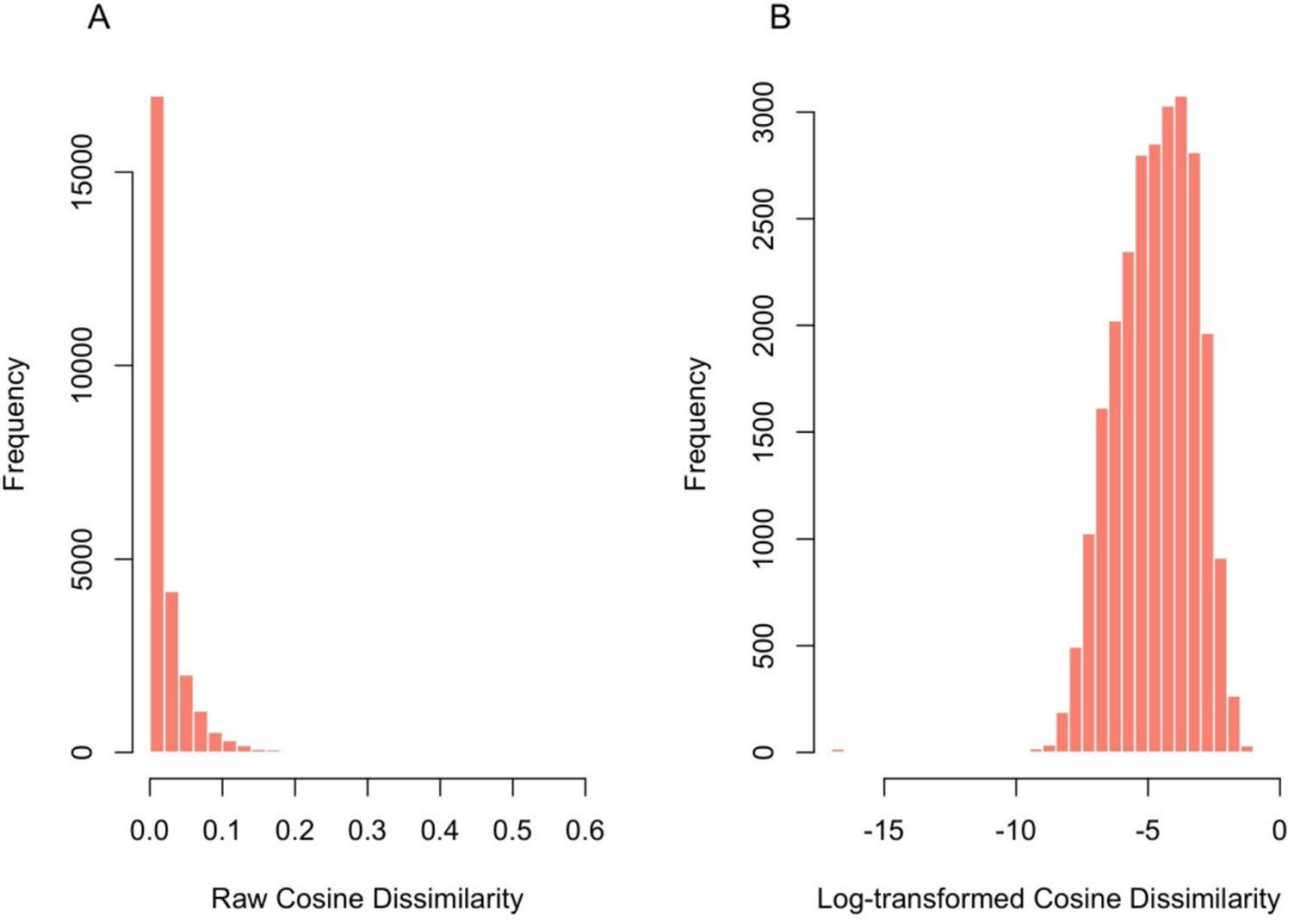
Distribution of informativeness values, before (**A**) and after (**B**) log-transformation

As a representative example, Fig. 1 shows dissimilarity estimates and informativeness scores before and after the log-transformation on the English materials of MECO-L1, showing that indeed what was a highly skewed distribution in raw values (panel A) becomes much more normal (panel B). In all analyses below, we therefore use the log-transformed value as our informativeness metric. Note that the log-transformation does not change the directionality of the measure: That is, in log-transformed values, less negative values (i.e., values closer to zero) reflect higher informativeness, whereas more negative values (i.e., further from zero) reflect lower informativeness (see examples, below).

### Data and code availability

The full analysis script is available via the Open Science Framework (OSF) website, at: https://osf.io/rh85d/. The repository also includes a downloadable Informativeness package, along with documentation, offering instructions for users interested in estimating informativeness for their materials.

## Results

### Basic properties of the informativeness measure: Examples and descriptive statistics

We first note that the proposed informativeness metric is context-specific, meaning that the same word can receive different values depending on the context in which it appears. To illustrate this feature, consider the word “wedding” in the two aforementioned sentences: 1) “the wedding was ruined by the heavy rain and snow” and 2) “the royal couple married in a beautiful wedding ceremony”. In the first sentence, the informativeness value of the word “wedding” is – 1.69. In contrast, in the second sentence, the informativeness value of “wedding” is lower, – 4.21. Likewise, the word “ran” is less important for the full meaning of the sentence “The athlete ran as she crossed the finish line of the marathon” (an informativeness value of “ran” is – 3.43), than in “The bride ran as she saw the groom in the wedding” (informativeness value of “ran” is – 2.31). Again, this is intuitively plausible – in the second sentence, the word “ran” describes a crucial part of the sentence’s message. These examples highlight how the current informativeness metric captures the varying importance of words within different sentence contexts.

**Fig. 2:**
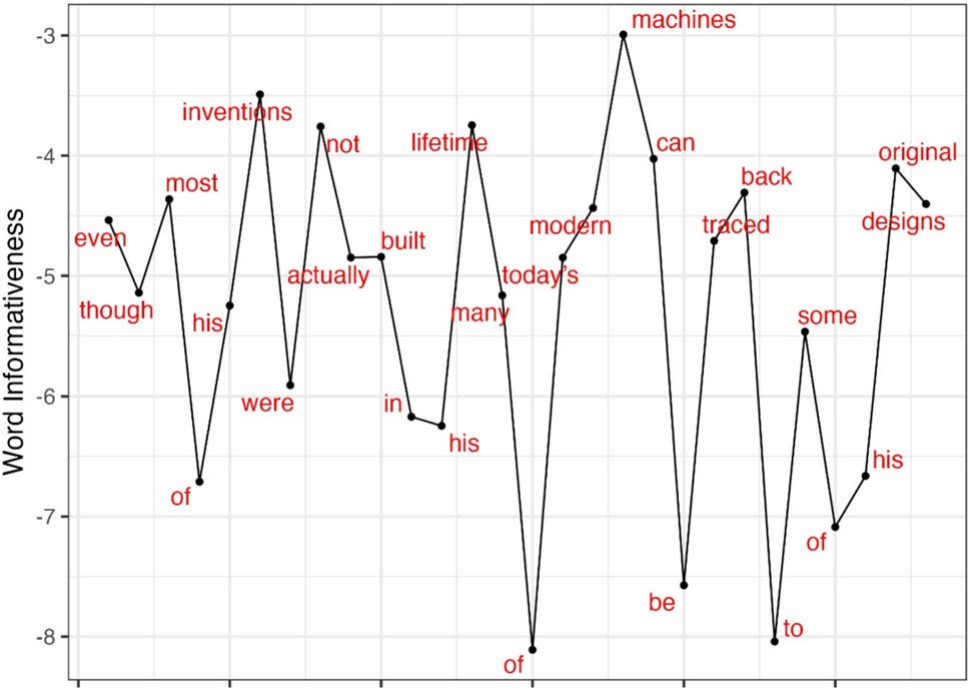
Informativeness values in an example sentence

To further examine the characteristics of the informativeness metric, Fig. 2 presents the informativeness values for each word in one sentence from the MECO-L2 database: “even though most of his inventions were not actually built in his lifetime, many of today’s modern machines can be traced back to some of his original designs”, and Table 1 presents the values that go into the calculation of the informativeness measure by word (the cosine similarity between the full and partial sentence; the dissimilarity metric; and the final log-transformed measure). The values presented in the figure and table demonstrate that certain words, such as “inventions” and “machines”, possess higher informativeness values, indicating their greater importance in conveying the full meaning of the sentence. Conversely, words like “of” and “be” exhibit lower informativeness values, suggesting they contribute less to the sentence’s overall information. In general, function words seem to contribute less information to the full sentence than content words, as expected. However, there is substantial variability within both content and function words, given their specific properties and the properties of the sentence.

**Table 1:** Values pertaining to the calculation of the informativeness metric in an example sentence

Word	Cosine similarity: full vs. partial sentence	Raw dissimilarity (1 minus cosine)	Informativeness: log-transformed dissimilarity
even	0.990	0.010	– 4.563
though	0.994	0.006	– 5.150
most	0.987	0.013	– 4.346
of	0.999	0.001	– 6.698
his	0.995	0.005	– 5.329
inventions	0.971	0.029	– 3.542
were	0.997	0.003	– 5.801
not	0.976	0.024	– 3.745
actually	0.992	0.008	– 4.864
built	0.992	0.008	– 4.833
in	0.998	0.002	– 6.276
his	0.998	0.002	– 6.229
lifetime	0.975	0.025	– 3.692
many	0.994	0.006	– 5.042
of	>0.999	< 0.001	– 8.262
today’s	0.994	0.006	– 5.041
modern	0.988	0.012	– 4.420
machines	0.949	0.051	– 2.976
can	0.982	0.018	– 4.025
be	0.999	0.001	– 7.511
traced	0.991	0.009	– 4.747
back	0.986	0.014	– 4.299
to	>0.999	< 0.001	– 8.017
some	0.996	0.004	– 5.489
of	0.999	0.001	– 7.260
his	0.999	0.001	– 6.748
original	0.984	0.016	– 4.139
designs	0.987	0.013	– 4.356

**Fig. 3:**
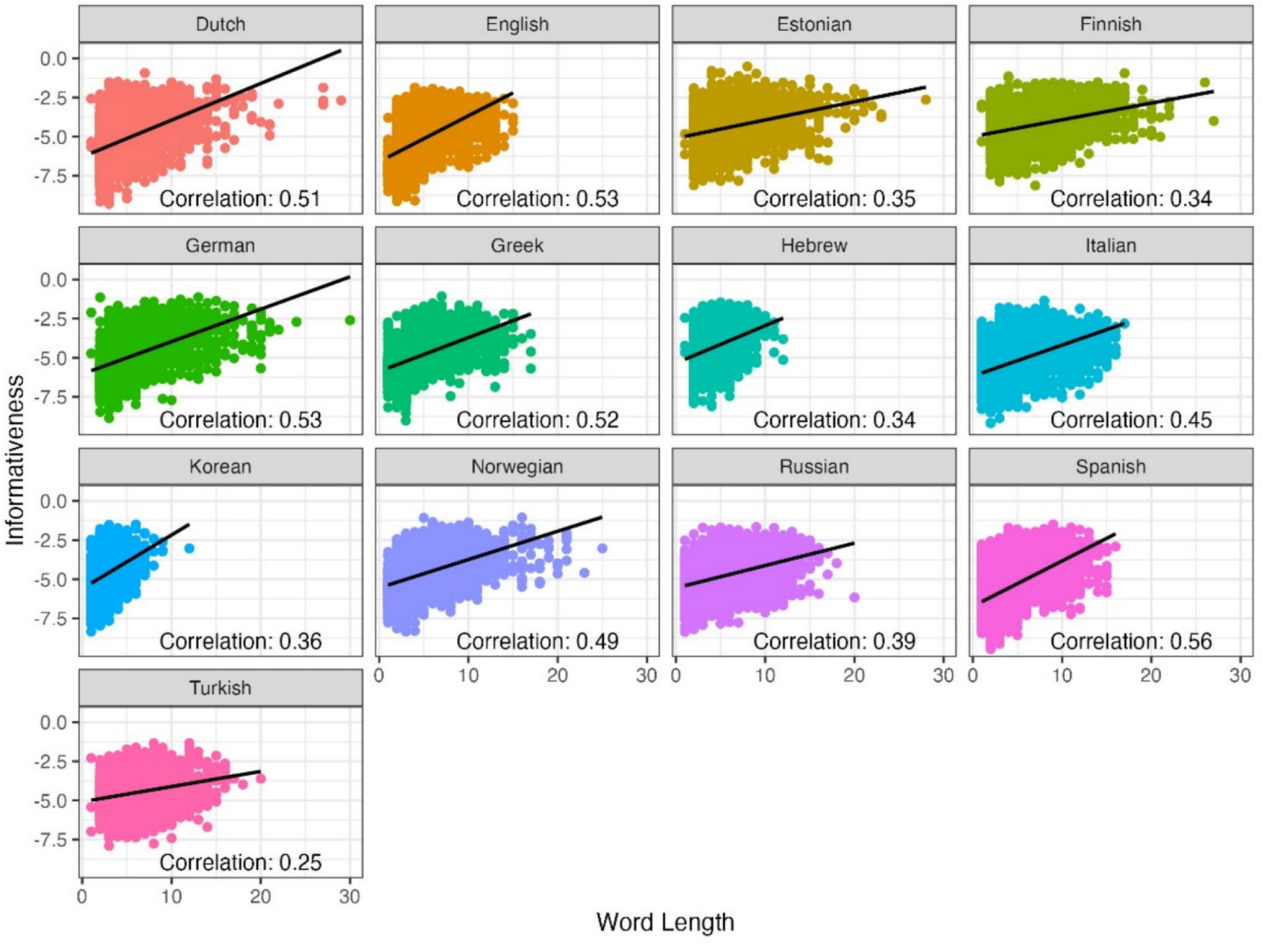
The relationship between informativeness and word length, in 13 languages

**Fig. 4:**
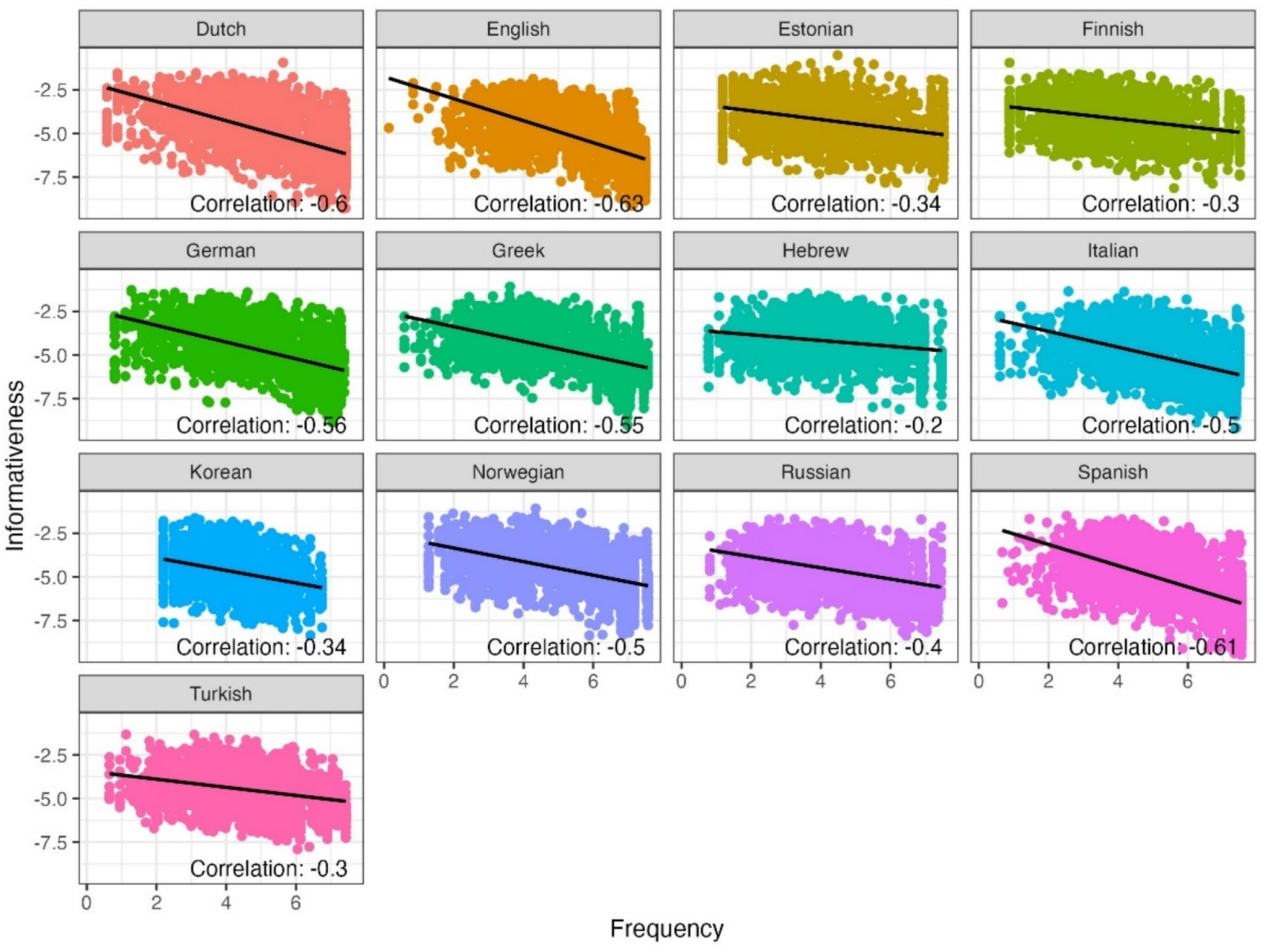
The relationship between informativeness and word Zipf-transformed frequency, in 13 languages

**Fig. 5:**
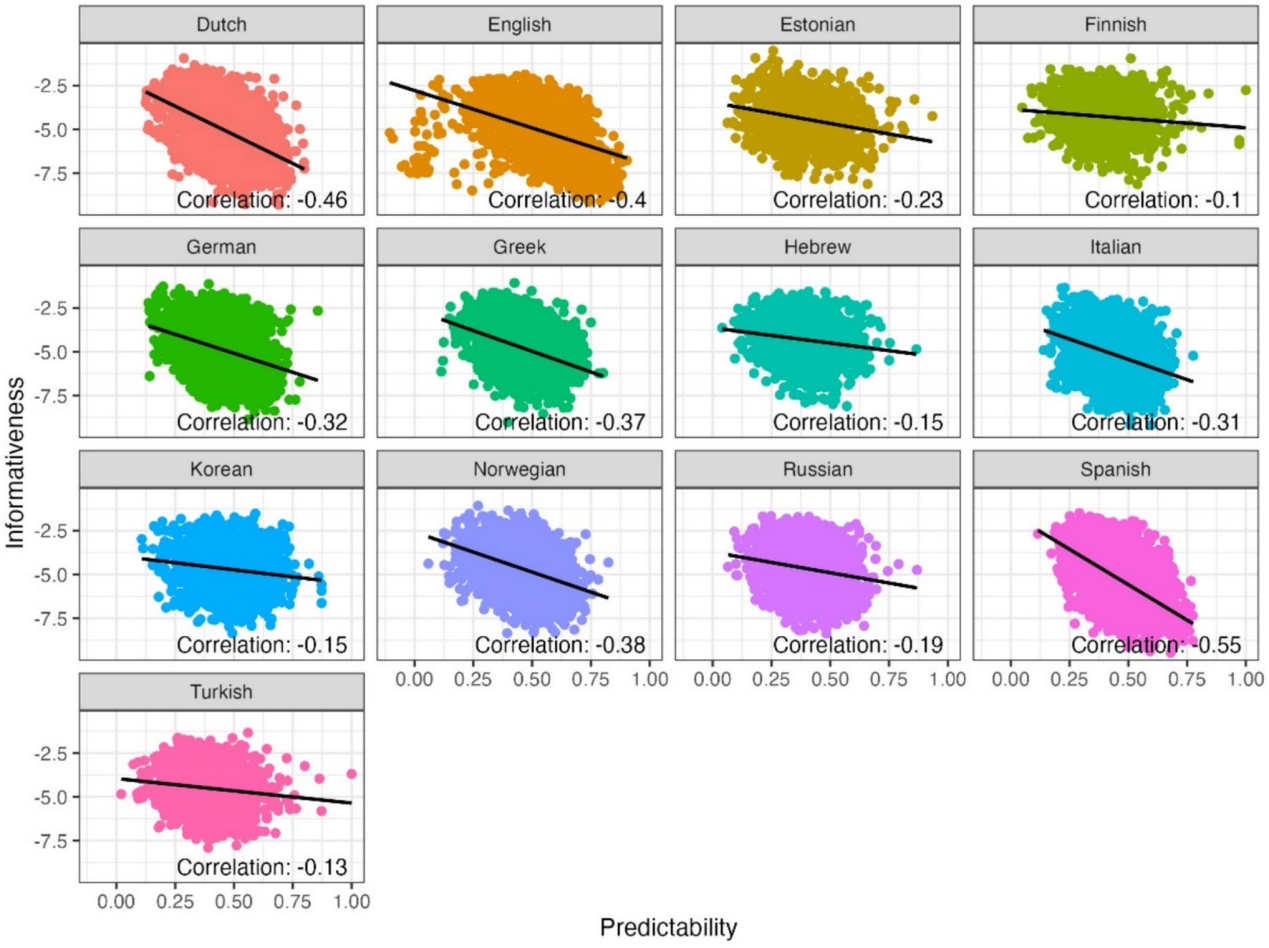
The relationship between informativeness and word predictability, in 13 languages

Another expected finding observed via this example is that shorter, more frequent and more predictable words generally seem to have lower informativeness values. To examine this more systematically, we next used all 12 texts in the MECO L1 corpus and computed the correlation between the informativeness metric and word length, Zipf-transformed frequency (i.e., log_10_(frequency per billion); Van Heuven et al., 2014) and predictability, in the 13 different languages (see Figs. 3, 4, and 5 for correlations with length, Zipf-transformed frequency and predictability, respectively). Based on visual inspection of these scatter plots, we excluded from our analysis informativeness values smaller than – 10, which formed clear outliers in these links and most likely have to do with sub-optimal estimation in the vectorial models (less than 0.1% of words in the texts). We observed a positive correlation between the informativeness metric and word length, showing that indeed, on average, longer words tend to be more informative within their respective contexts (across languages). Similarly, less frequent words tend to exhibit higher informativeness values. In other words, words that occur less frequently in language usage are more likely to carry substantial informational content and contribute significantly to the overall meaning of the sentence. Finally, in terms of the correlation with predictability, we found that as expected, words that are less predictable (i.e., more surprising) tend to display higher informativeness values. Despite these correlations however, it is important to note that informativeness is not fully correlated with length, Zipf-transformed frequency nor predictability (correlation values ranging from 0.25 to 0.56 for length; from – 0.63 to – 0.2 for Zipf-transformed frequency, and from – 0.55 to – 0.1 for predictability). This opens the door for additional predictive value by the informativeness metric in addition to these well-established predictors of reading behavior.

**Fig. 6:**
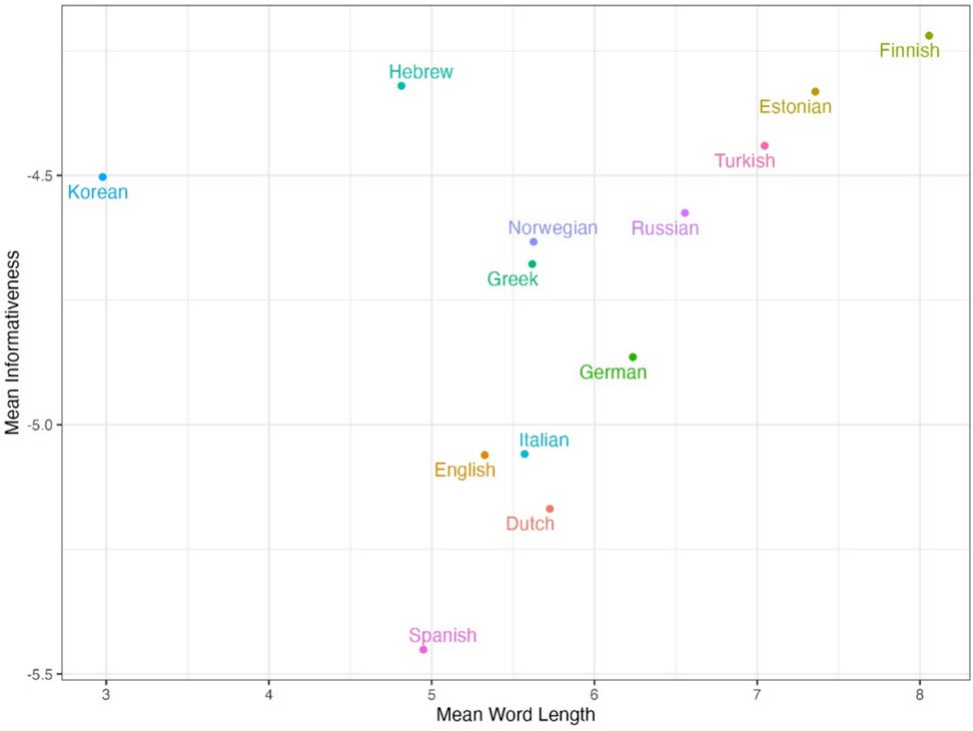
The relationship between mean informativeness values and mean word length, across 13 languages

Next, we computed for each language the mean informativeness level of words across a series of texts and compared them to the mean length of words in that language in the same texts. For this analysis, we only used the five translation-equivalent texts from MECO L1, to ensure that the texts are matched in their meaning across languages. We observed a clear and sensible relation between mean word length and mean informativeness values in a language: That is, words tend to be more informative on average in languages that use longer words (Fig. 6). This implies that languages with longer average word lengths have a greater capacity for expressing nuanced and detailed information within a single orthographic word (e.g., via more extensive affixation; note in particular the location of the agglutinative languages Finnish and Turkish in Fig. 5). The two clear exceptions to this trend were Korean, and, to a lesser extent, Hebrew, two languages that use shorter orthographic words but had relatively high mean word informativeness. However, this is expected when considering the different ways in which these two writing systems represent the *sounds* of the language (compared to the other languages included in MECO): In both languages, each orthographic character reflects multiple phonemes (in Hebrew due to its abjad script, where most letters represent CV syllables; and in Korean, which is based on syllabic printed units where components for the onset, nucleus and coda are visually represented by one character). Hence, the mean length of orthographic words in Hebrew and Korean most likely reflects an underestimation of their phonological word lengths, which are presumably more directly related to mean word informativeness.

**Fig. 7:**
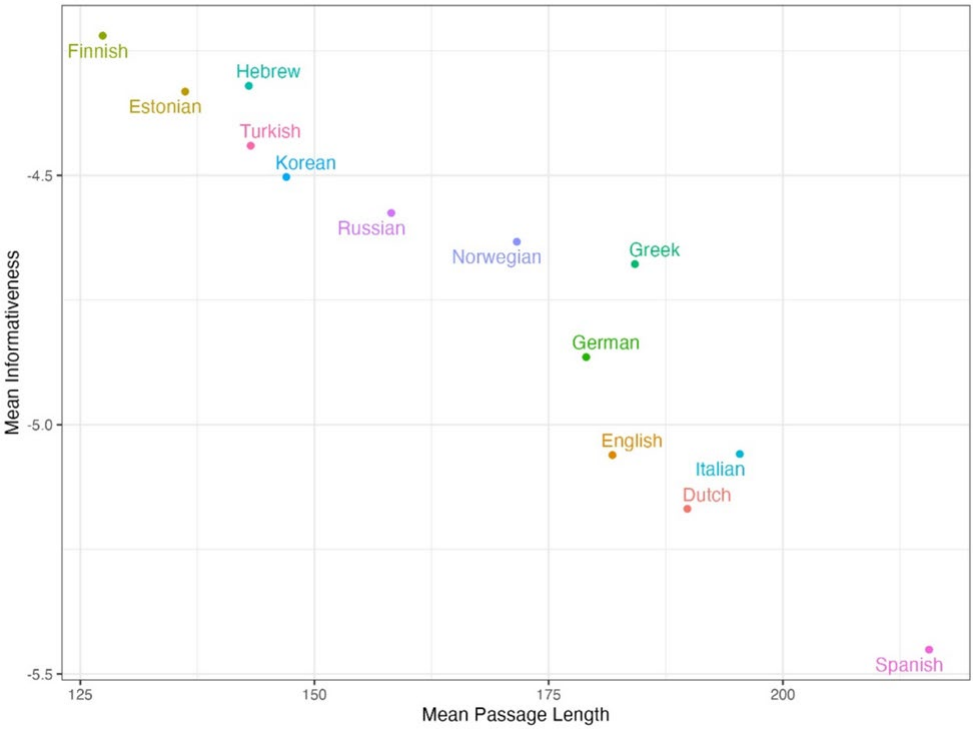
The relationship between mean informativeness values and mean passage length, across 13 languages

Another expected cross-linguistic pattern is the one between mean passage length (in words) and the mean informativeness value (Fig. 7; note that this analysis again included data only from the five translation-equivalent texts). As expected, we found that as the mean number of words in a passage increases, the corresponding mean informativeness value tends to decrease. This observation is again expected: On average, in languages that require fewer words to convey the same meaning, each individual word should convey more information (see Brysbaert, 2019a, for discussion). In contrast, languages that require more words to convey a given meaning should have a higher proportion of less informative words, resulting in a lower mean informativeness per word.

Finally, to provide an estimate of the convergent validity of our novel informativeness measure, we computed its correlation with a previous measure defined as the cosine similarity between the embeddings representing each word and the sentence in which it appears (including the word, see Fan and Reilly, 2020), in the 12 MECO-L1 texts in the 13 languages (note that for the purpose of the comparison semantic similarity was re-estimated using the same semantic space used in our definition). The details of this comparison are provided in Supplementary Materials S1; in a nutshell, we found that, as expected, there were positive correlations between the new definition and the previous definition used by Fan and Reilly (2020). However, these correlations were far from perfect (in the 0.5 to 0.65 range). This provides evidence for the convergent validity of our measure, while highlighting that, as expected, it does not fully overlap with previous definitions. For theoretical reasons outlined in the Introduction, we use our novel metric in the remainder of this paper. However, we invite interested readers to access the estimates using both approaches on the project’s OSF page (see Data and code availability).

### Validation using MECO-L1: Word informativeness effects across languages

In this section, we analyze the eye-tracking data from MECO L1. Our first goal is to validate the new measure by showing that it indeed has an impact on eye-movement behavior, and if so, to map the specific aspects of eye-movement that are most impacted by informativeness. Our second goal is to examine to what extent informativeness effects generalize or vary cross-linguistically.

To these aims, we employed a series of (linear and logistic) mixed-effect models. We used five measures as dependent variables reflecting different processing stages of eye movement behavior, from early to later stages: (1) first run skipping (i.e., a binary measure, whether a word was skipped or not during the initial pass), and, for words that were fixated at least once: (2) first fixation duration (i.e., the duration of the initial fixation on the word; log-transformed), (3) gaze duration (i.e., summed duration of fixations on the word during the initial pass; log-transformed), (4) total fixation duration (i.e., cumulative duration time of all fixations on a specific word; log-transformed), and (5) rereading (i.e., a binary measure that indicates whether the participant’s gaze returned to a previously read word after the first pass). The crucial fixed effect of interest was the informativeness value of each word (scaled and centered). We also included fixed effects for language (a 13-level effect-coded variable), and the interaction between language and informativeness. Additionally, we controlled for word length (in printed characters), Zipf-transformed frequency, and predictability (all centered and scaled), as well as for the interactions between each one of these factors with language (to ensure that the informativeness by language interaction does not stem from collinearity with these covariates). The models included random intercepts for subjects and for words within a language (the maximal random effect model that converged; Barr et al., 2013). Due to convergence failures of logistic models, even with this relatively simple random effect structure, we used an approximate fit via linear optimization (i.e., setting nAGQ to 0 in R) in models with skipping and rereading as dependent variables. Models were fitted using the lme4 package in R (Bates, 2010), using the bobyqa optimizer. The lmerTest package (Kuznetsova et al., 2017) was used to estimate the significance of the fixed-effect coefficients, and the *car* package (Fox & Weisberg, 2018) was used to run chi-square significance tests for full terms. For dependent variables with a significant interaction between language and informativeness, we ran additional models on each language's data separately to estimate the by-language simple effect of informativeness (with the same random effect structure as above and fixed effects for length, Zipf-transformed frequency, predictability, and informativeness). In what follows, we review the five models for each dependent variable.

### Dependent variable: First run skipping

**Fig. 8:**
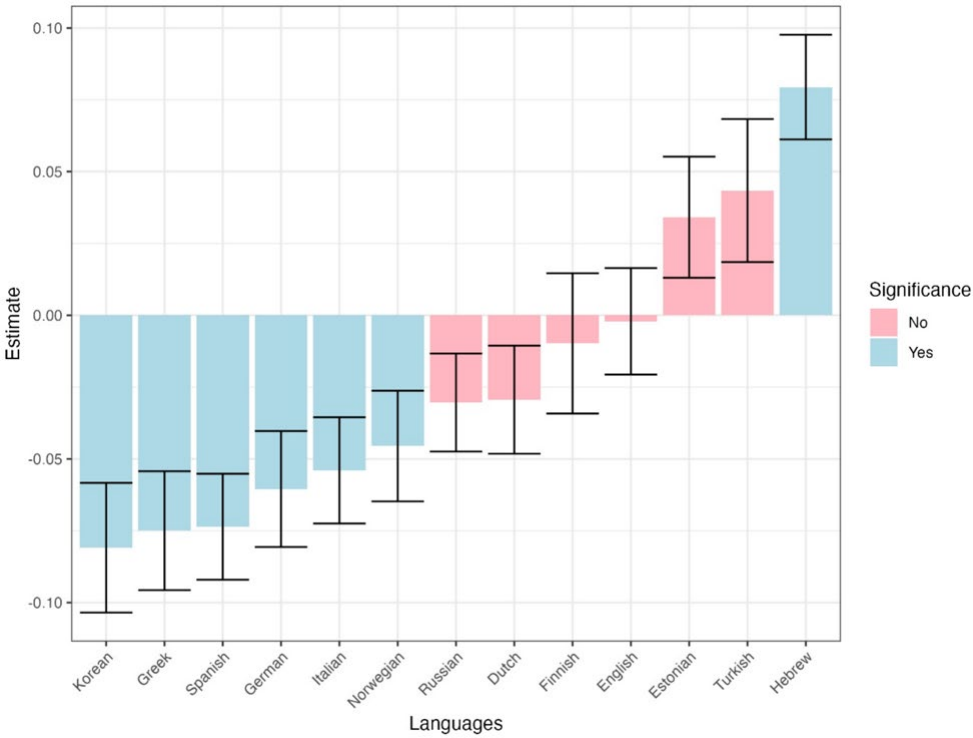
The effect of informativeness on first-run skipping across languages. *Errors bars* depict standard errors

Informativeness significantly influenced first run skipping, even after accounting for word length, Zipf-transformed frequency, predictability and overall differences in skipping across languages (Table 2), with less informative words skipped more often ($\beta =-0.02,p \mathrm{<} \, .001$). Moreover, there was a significant interaction between language and informativeness, indicating that the effect of informativeness on skipping differs between languages. Figure 8 displays the language-specific impact of informativeness on skipping, indicating that the effect of informativeness on skipping was significant in the expected direction in six out of the 13 languages, even when data from each language was considered separately. There was one case – Hebrew – where there was a “flipped” effect of informativeness on skipping (i.e., more informativeness words being skipped more often); however, given the lack of its theoretical interpretation and the large number of comparisons we presume that it reflects a type I error.

**Table 2:** Effects on first run skipping in the MECO L1 data

Predictor	β	se	Chisq	*df*	*p*
Word length	– 0.75	0.02	2331.46	1	$\mathrm{<}.001$
Zipf-transformed frequency	0.19	0.01	194.61	1	$\mathrm{<}.001$
Predictability	0.01	0.005	7.54	1	$.006$
Informativeness	– 0.02	0.006	11.29	1	$\mathrm{<}.001$
Language			103.63	12	$\mathrm{<}.001$
Word length × language			214.23	12	$\mathrm{<}.001$
Zipf-transformed frequency × language			36.73	12	$\mathrm{<}.001$
Predictability × language			51.41	12	$\mathrm{<}.001$
Informativeness × language			66.46	12	$\mathrm{<}.001$

### Dependent variable: First fixation duration

The analysis revealed that the informativeness metric did not significantly influence first fixation duration (Table 3). This suggests that the duration of the initial fixation on the word during the first pass only is not systematically impacted by word informativeness. The effect of the interaction between informativeness and language was also insignificant.

**Table 3:** Effects on first fixation duration in the MECO L1 data

Predictor	β	se	Chisq	*df*	*p*
Word length	– 0.003	0.002	3.03	1	$.08$
Zipf-transformed frequency	– 0.03	0.002	381.25	1	$\mathrm{<}.001$
Predictability	– 0.002	0.0008	5.71	1	$.017$
Informativeness	0.002	0.001	3.68	1	$.055$
Language			31.79	12	$.001$
Word length × language			17.79	12	$.12$
Zipf-transformed frequency × language			14.49	12	$.27$
Predictability × language			23.16	12	$.03$
Informativeness × language			17.11	12	$.14$

### Dependent variable: Gaze duration

**Fig. 9:**
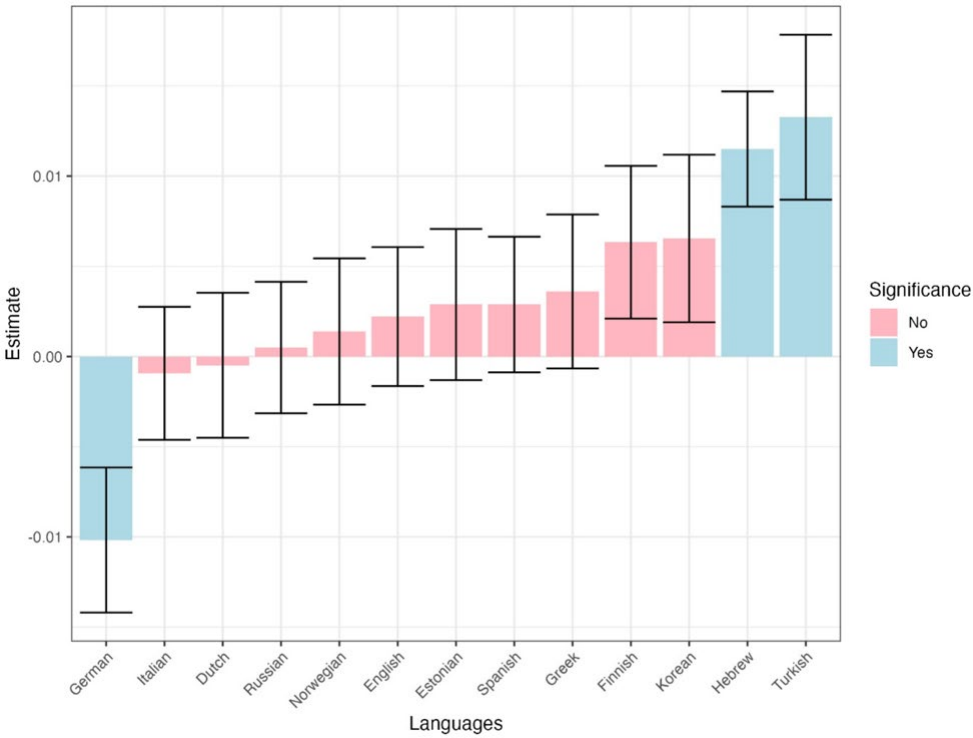
Effect of informativeness on gaze duration across languages. *Error bars* depict standard errors

Informativeness significantly influenced gaze duration, even after accounting for the control variables (Table 4), with longer gaze duration to more informative words ($\beta \mathrm{=} \, 0.004,p \mathrm{=} \, .002$). This suggests that in contrast to the more initial measure of first fixation duration, gaze duration, the summed duration of fixations on the word during the initial pass time, is indeed impacted by word informativeness. Moreover, there was a significant interaction between language and informativeness, indicating that the effect of informativeness on gaze duration differs between languages. However, as displayed in Fig. 9, the language-specific impact of informativeness on gaze duration was significant only in a limited number of languages (only two out of 13 languages showing a positive expected effect).

**Table 4:** Effects on gaze duration in the MECO L1 data

Predictor	β	se	Chisq	*df*	*p*
Word length	0.12	0.003	194.51	1	$\mathrm{<}.001$
Zipf-transformed frequency	– 0.06	0.002	591.61	1	$\mathrm{<}.001$
Predictability	– 0.008	0.001	62.46	1	$\mathrm{<}.001$
Informativeness	0.004	0.001	9.26	1	$.002$
Language			50.71	12	$\mathrm{<}.001$
Word length × language			82.47	12	$\mathrm{<}.001$
Zipf-transformed frequency × language			28.53	12	$.004$
Predictability × language			43.50	12	$\mathrm{<}.001$
Informativeness × language			29.31	12	$.004$

### Dependent variable: Total fixation duration

**Fig. 10:**
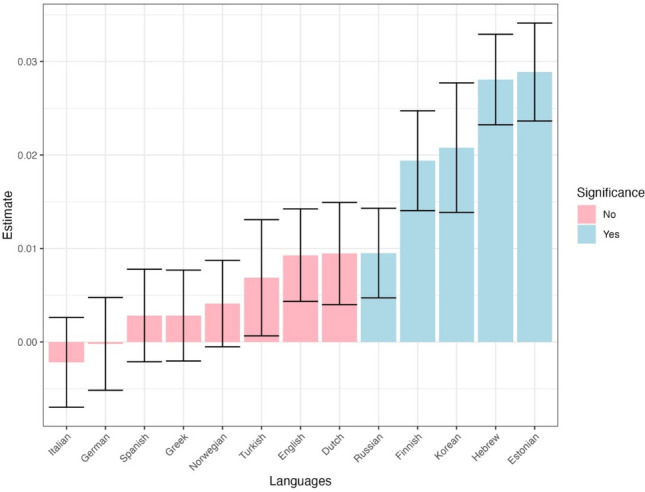
Effect of informativeness on total fixation duration across languages. *Errors bars* depict standard errors

Informativeness significantly influenced total fixation duration beyond the covariates (Table 5). Examining the models’ coefficient revealed that as expected, more informative words had longer total fixation durations ($\beta \mathrm{=} \, 0.012,p \mathrm{<} \, .001$). The interaction between language and informativeness was also significant, suggesting that the informativeness’ impact on fixation duration varies across languages (see Fig. 10 for the by-language estimates). This figure again highlights substantial potential cross-linguistic variability, with five out of the 13 languages demonstrating a significant impact of informativeness on fixation durations.

**Table 5:** Effects on total fixation duration in the MECO L1 data

Predictor	β	se	Chisq	*df*	*p*
Word length	0.15	0.003	1917.24	1	$\mathrm{<}.001$
Zipf-transformed frequency	– 0.01	0.003	993.44	1	$\mathrm{<}.001$
Predictability	– 0.02	0.001	242.85	1	$\mathrm{<}.001$
Informativeness	0.01	0.002	60.36	1	$\mathrm{<}.001$
Language			57.99	12	$\mathrm{<}.001$
Word length × language			57.16	12	$\mathrm{<}.001$
Zipf-transformed frequency × language			32.44	12	$.001$
Predictability × language			44.33	12	$\mathrm{<}.001$
Informativeness × language			57.63	12	$\mathrm{<}.001$

### Dependent variable: Rereading

The analysis of fixation times above points to a potential dissociation between early durational measures (first fixation duration and gaze duration) and later cumulative fixation durations (i.e., total fixation times). In particular, the main effect of informativeness was absent in first fixation duration, showing only limited effects in predicting gaze duration, but more pronouncedly shown in analyses of cumulative fixation times. In other words, it seems that as the reading process unfolds, the influence of word informativeness on durational measures becomes more evident. A potential candidate that can explain this dissociation may be the impact of informativeness on rereading – if more informative words are more prone to rereading (i.e., further fixations in subsequent runs), this can explain why the effect of informativeness was more clearly observed in total fixation times compared to earlier measures.

**Fig. 11:**
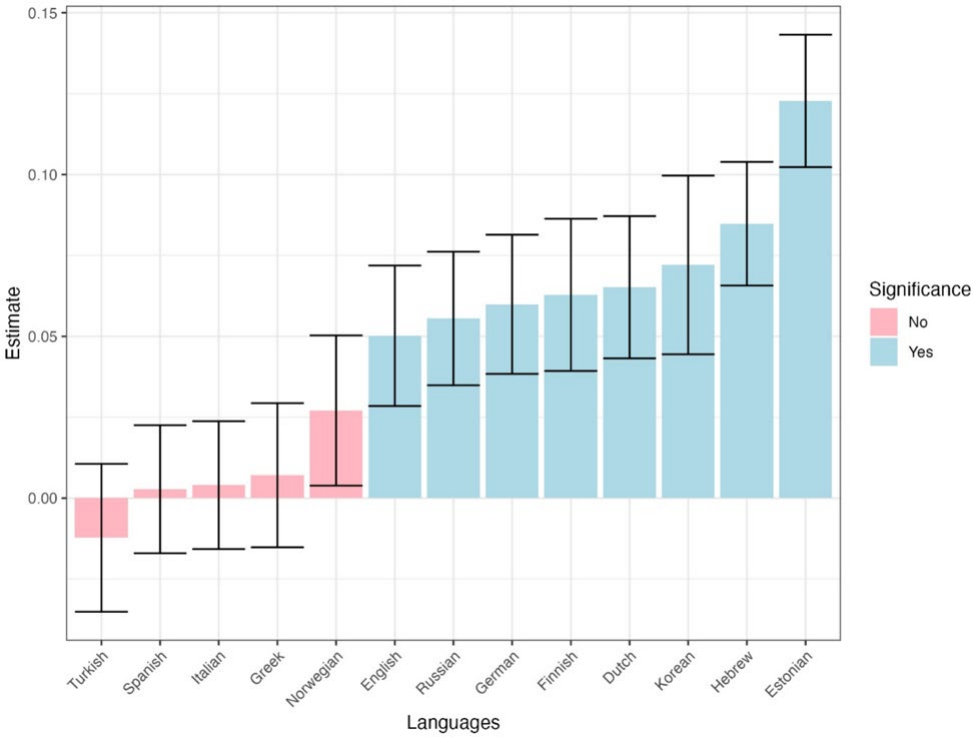
Effect of informativeness on rereading across languages. *Error bars* depict standard errors

In support of this prediction, we found that informativeness significantly impacted rereading probability after accounting for all control variables (Table 6), with more informative words more prone to rereading, as expected ($\beta \mathrm{=} \, 0.05,p \mathrm{<} \, .001$). The interaction between language and informativeness was also significant. Examining the by-language estimates (Fig. 11) shows a significant effect at the single language level in eight out of the 13, with the by-language estimate pattern mirroring, to a substantial extent, the one found for total fixation times (i.e., all five languages showing significant effects on total reading times also had significant effects on rereading probability). This further suggests that the effect of total reading times stems, at least to some extent, from an effect on rereading probability, where more important words are being reread more often.

**Table 6:** Effects on rereading in the MECO L1 data

Predictor	β	se	Chisq	*df*	*p*
Word length	0.17	0.01	170.63	1	$\mathrm{<}.001$
Zipf-transformed frequency	– 0.21	0.01	341.41	1	$\mathrm{<}.001$
Predictability	– 0.08	0.006	203.63	1	$\mathrm{<}.001$
Informativeness	0.05	0.007	56.73	1	$\mathrm{<}.001$
Language			86.74	12	$\mathrm{<}.001$
Word length × language			43.78	12	$\mathrm{<}.001$
Zipf-transformed frequency × language			18.46	12	$.102$
Predictability × language			39.61	12	$\mathrm{<}.001$
Informativeness × language			42.63	12	$\mathrm{<}.001$

### Summary: Effects of informativeness across languages in MECO-L1

**Fig. 12:**
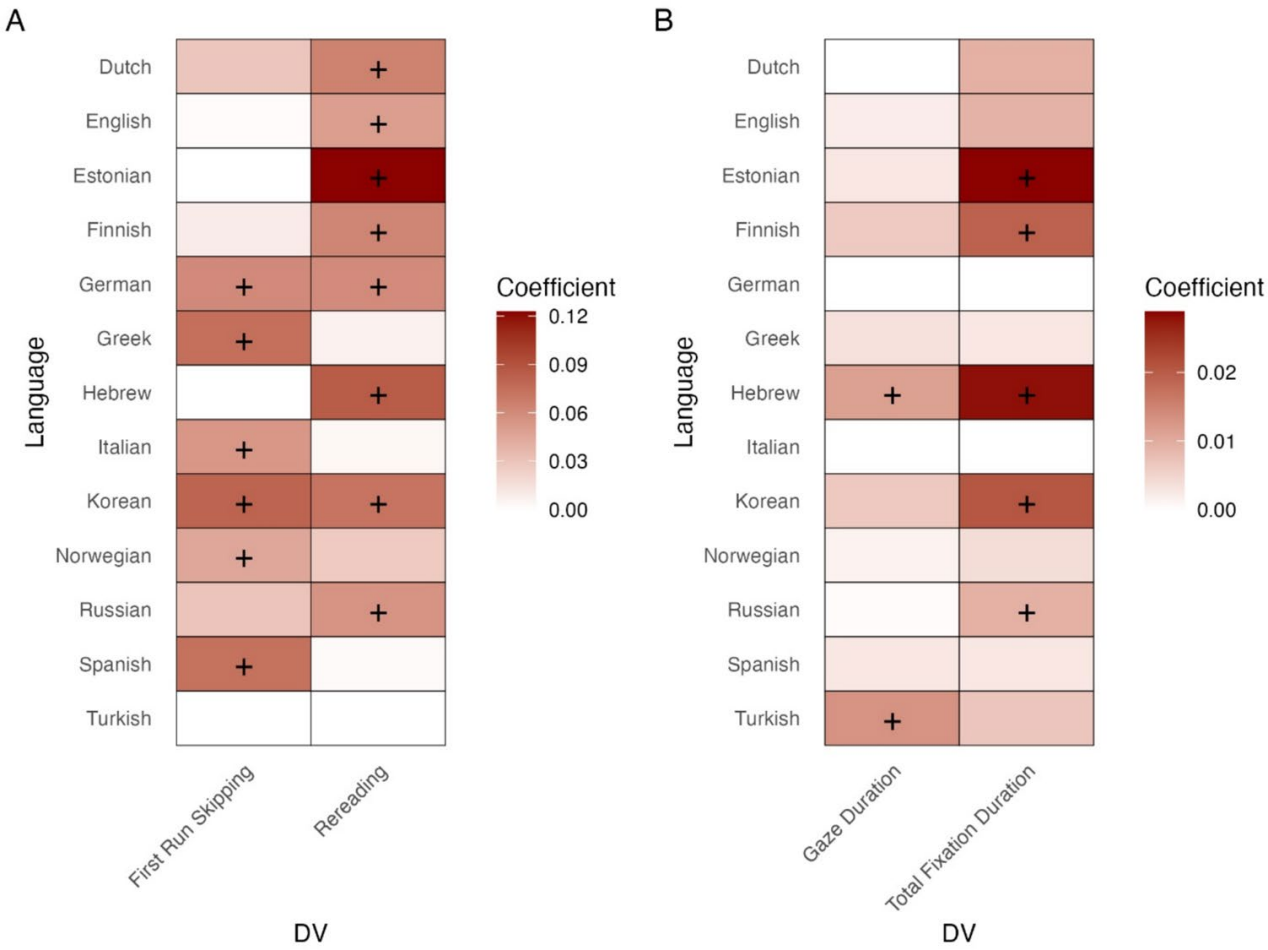
Summary of the effects of informativeness on each dependent variable across the MECO-L1 languages: Standardized coefficients from mixed-effect models. Binary measures and numeric outcomes are plotted separately given their different scales. **A** Binary outcomes; **B** Numeric outcomes. Note: The color intensity of each cell reflects the magnitude of the effect. Effects in the unexpected direction were zeroed out and appear as white cells for readability. Cells marked with a plus sign are significant (in the expected direction)

Overall, the analyses thus far show that our measure of informativeness does indeed have an impact on multiple aspects of eye-movement behavior, with significant main effects of informativeness on first run skipping, gaze duration, total reading time, and likelihood of rereading. At the same time, there is also evidence for cross-linguistic variability, with models on the same four dependent variables also revealing significant interactions between language and informativeness. To summarize the effects of informativeness across languages, Fig. 12 presents a heatmap illustrating the magnitude and significance of effects on these four dependent variables in each MECO-L1 language (as estimated via the separate by-language models). We briefly note at this stage that this pattern suggests a combination of cross-linguistic similarities (with informativeness affecting at least one dependent variable in all languages) and differences (informativeness often affecting different dependent variables across languages). We return to these cross-linguistic similarities and differences in the General Discussion, below.

#### Validation analyses using MECO-L2: Interactions with reading skill

We proceeded with our analysis by examining the effects of informativeness in the MECO-L2 data. As noted above, MECO-L2 includes eye-movement data from English only, which was the L2 for the majority of participants (11 out of the 12 included testing sites). Further, L2 readers in this sample varied substantially in their English skills, both within and across sites (see Kuperman et al., 2023, for details).

These features of MECO-L2 allowed us to achieve two additional goals. The first goal was to examine whether informativeness affects reading behavior above and beyond another potential covariate: words’ overall predictability as reflected in its surprisal. Thus, with all participants reading in the same target language (i.e., English), we could easily add the word’s surprisal (i.e., -log(probability)), as defined by its predictability in context, and quantified by the large language model generative pre-trained Transformer 2 (GPT-2; for similar usages of the same model to quantify predictability see, e.g., Cevoli et al., 2022; Shain, 2024). This inclusion is important for two reasons: First, informativeness and surprisal are positively correlated (e.g., in the MECO-L2 texts: *r* = 0.29), with more predictable words tending to be less informative. Second, and relatedly, surprisal from large language models encompasses multiple factors, including, in particular, syntactic predictability (see, e.g., Lopes Rego et al., 2024), which in turn could also be related to a word’s informativeness^3^. By controlling for surprisal, along with other covariates above, we examined whether informativeness has a unique effect beyond these factors.

The second goal was to examine how individuals with different reading skills in the target language (in this case, English) are impacted by informativeness. As noted in the Introduction, previous studies have suggested that centrality effects on real-time ocular measures tend to be greater in less proficient readers (Brandl & Hollenstein, 2022; Jayes et al., 2022; Lev, 2020; Schifer, 2019; Yeari et al., 2018); we expected to find a similar pattern when using our new measure of word informativeness.

To analyze the MECO-L2 eye-tracking data, we again used a series of (linear and logistic) mixed-effect models, similar to the ones used for the L1 analysis, while also using the individual differences data collected in MECO-L2 to examine whether reading skills in English qualifies the link between informativeness and eye-movement measures. Specifically, we computed a composite score for each participant by taking the mean of their *Z*-scores across the five English individual differences tests in MECO-L2 (i.e., TOWRE words and nonwords; spelling score; LexTALE accuracy; and vocabulary knowledge) and examined whether it interacts with informativeness in impacting eye-movement measures. All models on the MECO L2 data used a model similar to the one conducted in the MECO L1 analysis, testing the impact of informativeness on one dependent variable, with additional fixed effects for word’s surprisal, the composite score (centered and scaled), and the interaction between the composite score with informativeness, and controlling for the interactions between the reading skill composite with word length, log-transformed frequency, semantic predictability, and surprisal, to ensure that observed interactions with informativeness are not due to collinearity with these measures. Additionally, we controlled for site (a 13-level effect-coded variable), to account for potential overall differences between sites in the dependent variable.

In our full analyses of the MECO-L2 data, we considered the same five dependent variables as those analyzed in MECO-L1: First run skipping, first fixation duration, gaze duration, total fixation duration, and rereading. However, in the MECO L2 analyses, in all three earlier measures – first run skipping, first fixation duration, and gaze duration – there was no evidence for the hypothesized effects of informativeness on the dependent variable. For brevity, we therefore report the full analyses of these variables in Supplementary Materials S2, and here focus on the two dependent variables showing the more robust effects of informativeness (in the L1 and L2 data alike): Total fixation duration and rereading probability (we return to the variation between measures in the General discussion, below).

#### Dependent variable: Total fixation duration

**Fig. 13:**
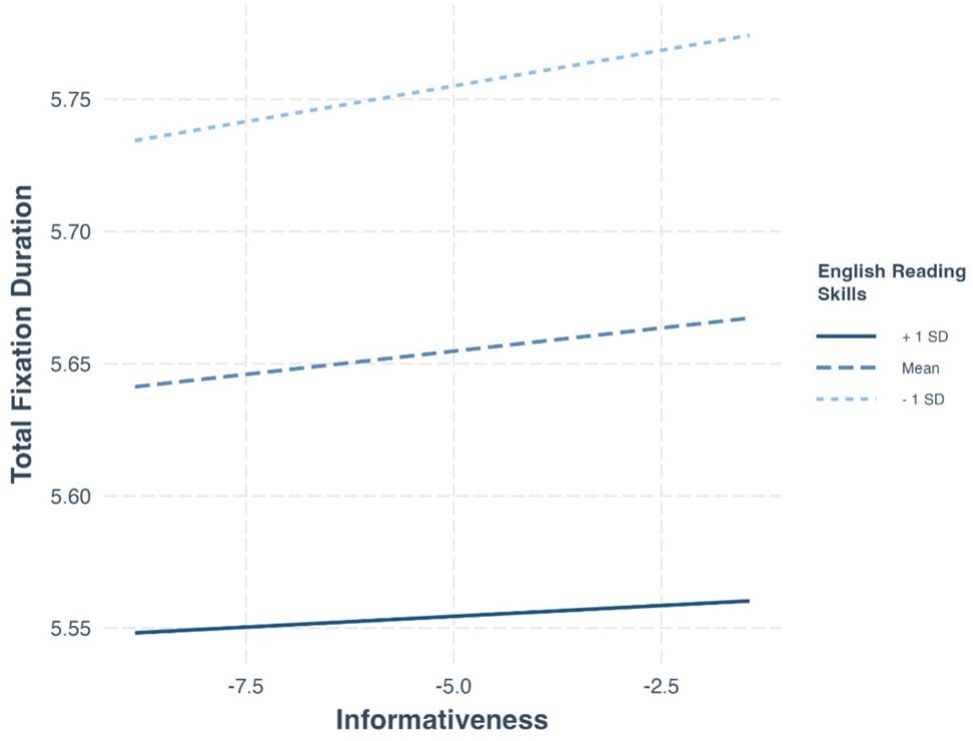
The interaction between informativeness and composite score in predicting total fixation duration

Replicating the results observed in L1 reading, informativeness had a positive effect on total fixation duration beyond all other included variables (Table 7;$\beta =0.005,p< \, .001$). Importantly, there was a significant interaction between informativeness and the composite reading score ($\beta =-0.003,p=0.002$), suggesting that the influence of informativeness on reading duration differs across readers with different levels of reading skill. Specifically, Fig. 13 illustrates that whereas there is a positive slope of informativeness on fixation duration across levels of reading proficiency, less proficient readers are characterized by a *steeper* slope. This suggests that, as expected, the impact of informativeness on fixation duration is more pronounced for individuals with lower reading skills compared to more skilled readers.

**Table 7:** Effects on total fixation duration in the MECO L2 data

Predictor	β	se	Chisq	*df*	*p*
Word length	0.14	0.006	496.06	1	
Log-transformed frequency	– 0.06	0.006	86.27	1	
Predictability	– 0.005	0.001	12.85	1	
Surprisal	0.05	0.001	120.51	1	
Informativeness	0.005	0.002	12.24	1	
Site			48.67	11	
Composite score	– 0.10	0.009	119.00	1	
Composite score × Word length	– 0.02	0.001	431.21	1	
Composite score × Log-transformed frequency	0.008	0.001	49.89	1	
Composite score × Predictability	0.002	0.0009	6.68	1	
Composite score × Surprisal	– 0.003	0.0009	13.74	1	
Composite score × Informativeness	– 0.003	0.0009	10.76	1

#### Dependent variable: Rereading

**Fig. 14:**
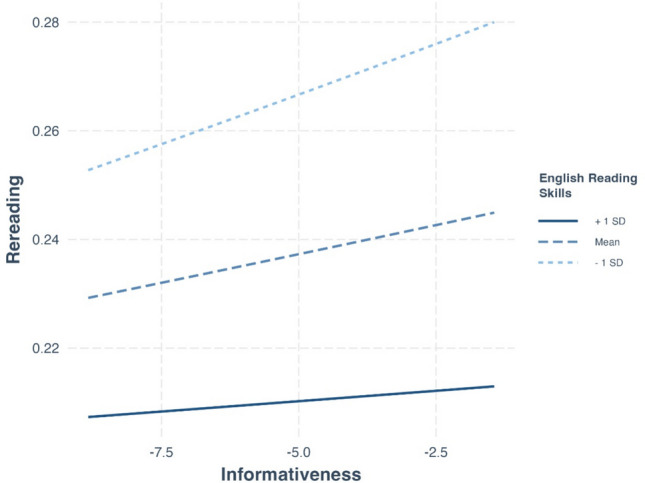
The interaction between informativeness and composite score in predicting rereading

As observed in L1, informativeness had a significant positive effect on rereading beyond all other included variables (Table 8; $\beta =0.016,p=.038$). Notably, we again observed a significant interaction between informativeness and the composite reading score ($\beta =-0.01,p \mathrm{=} \, .023$). Mirroring the interaction with reading skill found in total fixation time, it was the readers with the lower proficiency who were more impacted by informativeness in their rereading, as indicated by their steeper slope shown in Fig. 14. This finding again suggests that the impact of informativeness on eye-movement reading behavior is more pronounced for individuals with lower reading skills compared to more skilled readers, also in rereading probabilities.

**Table 8:** Effects on rereading in the MECO L2 data

Predictor	β	se	Chisq	*df*	*p*
Word length	0.17	0.02	61.41	1	
Log-transformed frequency	– 0.08	0.02	13.87	1	
Predictability	– 0.02	0.007	5.39	1	
Surprisal	0.18	0.007	667.18	1	
Informativeness	0.02	0.008	5.63	1	
Site			90.78	11	
Composite score	– 0.16	0.03	22.65	1	
Composite score × Word length	0.05	0.005	116.59	1	
Composite score × Log-transformed frequency	0.01	0.005	4.43	1	
Composite score × Predictability	0.01	0.005	5.43	1	
Composite score × Surprisal	0.01	0.004	12.11	1	
Composite score × Informativeness	−0.01	0.005	6.19	1

## General discussion

The main goal of this paper is to introduce a novel measure for assessing the significance of a word to the meaning of the sentence in which it appears, which we refer to as a word’s “informativeness”. This measure, we argue, adds an important tool for reading researchers as it offers an objective, interpretable, and language-general quantification. As such, our measure offers a computational and conceptual alternative to available approaches: Unlike common measures of centrality, which often rely on manual and subjective judgments, our operationalization offers an “automatic” approach; and unlike previously suggested computational measures, our quantification leverages advances in DSMs while maintaining interpretability via the simple method of comparing embeddings of sentences with and without a given word.

After showing that our new measure presents expected behavior in terms of its relation to psycholinguistic variables (e.g., word frequency, length, predictability, and cross-linguistic differences in mean word- and passage length), we ran a series of validation analyses against a large-scale dataset of eye-movement reading behavior. Our findings reveal that our new informativeness measure indeed significantly influences multiple aspects of eye-movement behavior (beyond known covariates): In particular, informativeness had stable effects on eye-movement measures that reflect later processing stages, namely, total fixation duration and rereading rate. Further, the main effects of informativeness on later eye-movement measures generalized to both first- and second-language readers and, to a substantial extent, to diverse writing systems. In contrast, there were more limited effects in some early eye-movement effects (e.g., some effects in first run skipping and gaze duration in the L1 data overall and in some languages therein, but no evidence for parallel effects in the L2). Furthermore, we found notable interactions between informativeness and language, and between informativeness and readers’ levels of reading skills. Generally, the results of the validation analyses are in line with previous accounts made based on other quantifications (e.g., on how centrality impacts reading behavior). However, they also further refine our understanding of how informativeness impacts reading behavior, and highlight open questions for future research. In what follows, we discuss these refinements and questions.

## The dynamics of the impact of informativeness on eye-movement behavior

Our analyses suggest that the effects of informativeness on eye movements stem from specific modulation of eye-movement behavior. Broadly speaking, the effect of informativeness seems to stabilize and become more evident later in the processing stream. Thus, we found that informativeness impacted first run skipping behavior in the L1 data on average (i.e., a significant main effect) – with more informative words being skipped less often – but this effect was absent from the English L2 data. Early durational measures – first fixation duration and gaze duration – showed little to no effects (e.g., main effect in gaze duration but only little evidence in individual languages when considered separately, and no evidence in the L2 data; and no evidence for corresponding effects on first fixation duration). In contrast, in cumulative reading time, informativeness seemed to have substantial effects (with longer reading times for more informative words, across multiple languages and in L1 and L2 data, similarly). Further analyses suggest that the dissociation between early and later durational measures originates at least in part from robust effects of informativeness on rereading probability, with readers tending to reread informative words more often.

Jointly, our interpretation of these findings is that readers’ sensitivity to word’s informativeness during reading spans both early and late processing stages. Thus, the main effect of informativeness in the L1 data on first run skipping rate suggests that readers can under some conditions “evaluate” (most likely implicitly, potentially also via partial processing of parafoveal information) whether an incoming word is central to the meaning of a larger written unit (in our case, sentences), and “decide” whether to skip it accordingly. However, once readers do fixate on a word in their early reading of it, informativeness has but limited effects on durational measures. Importantly, these early effects are then joined by a later, seemingly more robust, effect, which is that more informative words exhibit a higher tendency for rereading. The latter effect suggests that readers are able to revisit and reexamine informative words to better understand their content and extract additional meaning, potentially reflecting a more strategic behavior that aids comprehension.

The view that informativeness affects the reading process both via early and late mechanisms is consistent with the literature on the effects of predictability (Staub, 2015; 2024). On the one hand, there is a consensus that predictability can facilitate the reading process via an early lexical preactivation mechanism that affects word skipping and first fixation duration (Sheridan & Reingold, 2012). On the other hand, there is also ample evidence that predictability facilitates the integration of information into the existing discourse representation at a later post-lexical stage (e.g., Luke & Christianson, 2016; Sereno et al., 2018). Informativeness seems to follow a similar trajectory, but with particularly important (and cross-linguistically stable) post-lexical effects. This time course is also consistent with studies on centrality, which have shown that centrality mainly affects later reading measures (e.g., Hyönä & Niemi, 1990; Jayes et al., 2022).

In terms of implementing the effect of informativeness in computational models of reading, it seems therefore that informativeness most likely affects the post-lexical integration stage *I*, which was introduced in version 10 of the E-Z Reader model (Reichle et al., 2009) and is assumed to reflect the integration of word *n* into the existing discourse representation. If the integration of a word fails, a regressive saccade to the location where the difficulty became evident is programmed, and a rereading sequence is initiated. In its present form, the E-Z Reader model assumes that only predictability (indirectly) affects the probability of reprocessing, as words that are less predictable are harder to integrate. The present findings, by contrast, suggest that informativeness has an additional and potentially more direct effect on the integration phase.

## Cross-linguistic similarities and differences

We started our analyses by exploring the distribution of informativeness values across languages, to examine the interplay between a writing system’s characteristics and the informativeness of its words. We found a clear positive association between mean word length and mean informativeness values. This shows that languages with longer average word lengths “pack” more information in each word. We further observed a negative relationship between mean passage length (for translated passages) and mean informativeness values: In languages that require more words to convey the same meaning, each individual word is less informative on average. Together, this descriptive analysis showed that longer words permit a higher information density, while the use of fewer words to convey a message results in a “denser” delivery of information (and see Brysbaert, 2019a, for a related discussion).

However, the estimated effects of informativeness on eye-movement behavior across languages pointed to both similarities and differences. In terms of similarities, we found evidence for an effect of informativeness in diverse languages (e.g., Fig. 11 above showing effects on rereading in the typologically very different languages Dutch, Hebrew, and Korean). To clarify, this effect was present even when data from each of the languages were considered separately, despite the relatively low statistical power of such a by-language analyses (each MECO site had less than *N* = 50 participants which leads most likely to underpowered analyses, Brysbaert, 2019b; see also Siegelman et al., 2022a, b, for a detailed discussion of power in MECO). This shows the potentially ubiquitous nature of informativeness effects when it comes to explaining patterns of eye-movement reading behavior across languages (see Hollenstein et al., 2021, for converging evidence, and see more below). At the same time, there was also evidence for cross-linguistic differences: In fact, in all models where we found a significant main effect for informativeness in the MECO L1 data, there were also significant interactions between informativeness and language.

To further inspect these cross-linguistic similarities and differences, we look back at the summary of the effects across languages in the MECO-L1 (Fig. 12 above). On the one hand, this Fig. again highlights the cross-linguistic generalizable nature of informativeness effects – in all languages there was a significant impact of informativeness in at least one dependent variable, and in 12 out of the 13 languages there was evidence for an effect of informativeness on either rereading or first run skipping, which are seemingly most influenced by this metric (again, despite the relatively low power in by-language analyses). In fact, the only writing system where no effect was found in first run skipping or rereading was Turkish, which includes a smaller sample size in MECO (*N* = 29, compared to *N* = 45–50 in other sites, see Siegelman et al., 2022a, b). Another potentially interesting finding is that the effects on rereading and first run skipping, in particular, seem to be non-overlapping – in 2 of 13 writing systems only, Korean and German, there were significant effects of informativeness on both dependent variables. In the remaining ten languages, informativeness significantly impacted either of the two variables, but not both. This raises the intriguing possibility that readers in different languages generally adopt one of two strategies – either they are primarily impacted by informativeness in their initial “decisions” of whether to skip a word; or they rely on this source of information in subsequent reading of the text.

Clearly, what drives these cross-linguistic differences is an open question that requires further research and targeted experimentation. From a statistical learning perspective, these cross-linguistic differences can be interpreted as suggesting that in light of variation in the systematicity of various cues in a given written language, including the extent of the regularities between print, sound, and meaning, and the quasi-regular organization of the printed units in texts, readers in the language adopt a differential reliance on various available cues, including word-level informativeness (see Frost, 2012; Seidenberg, 2011; Smith et al., 2021, for related discussions). Which constraints would result in an increased or decreased reliance on word informativeness in different stages of reading, and furthermore, what interactions exist between informativeness and other sources of information within and across languages, are questions that we leave for future research.

## Individual differences in reliance on word informativeness during reading

The availability of multiple measures of individual differences in reading and language skills in the MECO L2 repository enabled us to examine how informativeness affects change as a function of English proficiency. The results were clear and generalized across dependent variables: The effects of informativeness on fixation duration and rereading were more prominent among individuals with lower reading skills compared to more skilled readers. This finding, which replicates and extends related previous findings (Brandl & Hollenstein, 2022; Jayes et al. 2022; Lev, 2020; Schifer, 2019; Yeari et al., 2018), suggests that while there appears to be a “centrality deficit” in offline measures of processing (i.e., reduced recall of central ideas for individuals with poorer reading skills), real-time measures of processing show that these individuals exhibit *increased* effort on words with high informativeness. Again, this may imply a compensatory strategy, wherein informativeness serves as a particularly important source of information for readers who may benefit from additional cues to access meaning from print.

At face value, this finding seems to go against existing studies on individual differences in statistical learning and reading, which often point to *increased* reliance on statistical and other cues during reading among *better* readers (see e.g., Siegelman et al., 2020, for evidence regarding reliance on O-P and O-S regularities). However, in this context, it is again important to remember that informativeness is only one of a multitude of quasi-regularities and cues that are available to readers in their writing system. Per this approach, good readers would be those who would increasingly rely specifically on more efficient regularities and cues to access meaning from print in the context of a given reading task. Thus, we posit that good readers rely less on informativeness because they can access the text’s meaning by relying on other, more immediate regularities (such as word-level regularities between print and meaning; see Siegelman, Rueckl, et al., 2022a, b). In fact, this is an idea that could be traced back to earlier notions of compensatory reliance on context in readers with poor word-level processing (Stanovich, 1980). To test this idea, future studies are left with further examining how readers at different skill levels are impacted by various cues, including informativeness, and how they differentially rely on different sources of information given their efficiency in a given text and context. Furthermore, an open question is whether and how informativeness interacts with other (e.g., word-level) regularities, and whether proficient readers increasingly rely on informativeness specifically when other cues are less available (see, e.g., Amenta et al., 2023; Strain & Herdman, 1999, for discussions in the context of other cues and regularities).

## Methodological notes and future possible modifications of the measure

First, it is noteworthy that in the current work, we only applied our approach to assessing the informativeness of words within sentences (using multilingual sentence-level embeddings from sentence-BERT). However, we maintain that our definition is theoretically extendable to other smaller-within-larger relation of text segments, pending developments of DSMs that will offer accurate embeddings at various sizes (e.g., sentences and passages). More broadly, it is important to note that in the current work, we have limited our quantification and validation to the informativeness of orthographic words, in alphabetic systems that clearly demarcate word boundaries. This was a decision driven primarily by practical consideration (i.e., the availability of large-scale eye-movement reading datasets of alphabetic writing systems with dependent variables defined at that level). However, it raises not only practical questions (e.g., how to demarcate words in unspaced writing systems such as Chinese; see Fan & Reiley, 2020), but also a theoretical question regarding the relevant *psychological* units whose informativeness leads to the observed effects. Thus, in alphabetic and other types of writing systems alike, it is possible that in some cases, readers are, in fact, firstly impacted not by the informativeness of each orthographic word, but rather that of larger units. Arguably, this is quite likely for idioms and other formulaic multiword units, but could also be the case in other constructions (e.g., the “give up” example from earlier – we have assumed that “up” is a relevant unit whose informativeness impacts behavior, but it is possible that the relevant unit is the full phrasal verb).

Second, we wish to clarify the senses in which our measure of informativeness is and is not context-dependent. It is context-dependent in the sense that it uses BERT’s contextual embeddings – that is, embeddings that provide different representations for the same orthographic unit in different sentential contexts (e.g., the word “bank” would be differentially represented in the context of “river bank” vs. “money bank”, thus impacting subsequent informativeness measures; cf. Fan & Reilly, 2020). Relatedly, our measure is also context-dependent in an important sense: the same word can have different informativeness values depending on the sentence in which it appears (see examples above). At the same time, our measure is *reading*-*task independent*. This is despite the fact that reading is a task-dependent process, and that there is evidence that what is considered “informative” or “central” may vary as a function of the reading task (Kaakinen et al., 2002; Van Der Schoot et al., 2008), and, furthermore, that centrality effects on eye movements vary as a function of the reading task at hand (Jayes et al. 2022; Yeari et al., 2015). How exactly to quantify informativeness in a way that takes into account a reader’s task, is a non-trivial question requiring future research.

Lastly, the examples above demonstrate that the factors underlying and influencing the informativeness of different printed units, and the scope of other potential constructs to which it relates, require further exploration. Arguably, informativeness is related both to factors we have mentioned above (e.g., semantic and syntactic predictability, surprisal more broadly), and additional constructs such as polysemy (e.g., consider the difference in informativeness of “bank” in “money bank” vs. “river bank”), and other aspects of morphological, syntactic, and discourse-level relations. In this current work, we have attempted to rule out some of these constructs as potential confounds, via statistical controls. Yet, clearly, understanding how informativeness is related to relations and constraints at different linguistic levels requires further work (and most likely, targeted experiments and not only corpus analyses). At a minimum, we believe that our validated quantification provides a useful measure that captures a complex psychological construct – the importance of a given printed unit to a larger unit in which it is embedded – and we encourage future research to continue to examine the factors that influence it as well as its unique contribution to reading behavior.

## Supplementary Information

Below is the link to the electronic supplementary material.

Supplementary file1 (DOCX 367 KB)

## Data Availability

Full data are available via the Open Science Framework (OSF) website: https://osf.io/rh85d/.

## Footnotes

1 Ideally, one may opt to use the same vectorial space to quantify both informativeness and predictability. However, the vectorial space we use for the informativeness measure was too noisy to provide reasonable measures of predictability based on short fragments of sentences (i.e., early in the sentence, where preceding context is of a small size). Following Bianchi et al. (2020), we therefore use *fastText* for word predictability instead.

2 Hypothetically, one could find values smaller than 0, if omitting a word from a sentence results a meaning *opposite* to the meaning of the full sentence. However, in practice, we did not observe such values in MECO texts.

3 Consider, for example, the word up in the two sentences: “I gave up on the project” vs. “I climbed up the ladder”. The word “up” is highly informative in the first sentence (value of – 0.68) but much less so in the second sentence (value of – 4.74). Arguably, however, the syntactic structure and predictability of these two constructions differ (the verb “climb” not needing a direction). Surprisal as quantified by large language models should encompass these types of relations and including it as a covariate should partial out their effect on behavior.

## Code availability

All code is available via the Open Science Framework (OSF) website: https://osf.io/rh85d/.

## Declarations

### Open practices statements

The full analysis code and data are available via the Open Science Foundation website, along with a downloadable and documented Informativeness package for users interested in estimating informativeness for their materials. See: https://osf.io/rh85d/. Because analyses uses previously publicly available data, no pre-registration was done.
