# Supplementary material for: Quantifying word informativeness and its impact on eye-movement reading behavior: Cross-linguistic variability and individual differences
Source: Behav Res Methods. 2025 Nov 11;57(12):343. doi: 10.3758/s13428-025-02878-x (PMC12605455; doi:10.3758/s13428-025-02878-x)
Supplement: Supplementary file 1 — Supplementary file1 (DOCX 367 KB) [file 13428_2025_2878_MOESM1_ESM.docx]

**Supplementary Materials for *Quantifying Word Informativeness and Its Impact on Eye-Movement Reading Behavior: Cross-Linguistic Variability and Individual Differences*.**

**Supplementary Materials S1: The relation between the new informativeness measure and Fan and Reilly’s previous operationalization.**

In this section we examine the relation between our novel operationalization of word informativeness and that provided by Fan and Reilly (2020). First, to produce the measure by Fan and Reilly, we examined the semantic similarity using the cosine of the angle between the embeddings representing each word and its containing sentence. For that we used the same pre-trained versions of sentence-BERT from the Python framework *sentence-transformer* (Reimers & Gurevych, 2019), utilizing the multilingual pre-trained model *distiluse-base-multilingual-cased-v2v* (Reimers & Gurevych, 2019, 2020); i.e., we used the same vectorial space for this previous operationalization as the one used for our informativeness measure.

Figure S1 shows the correlations between the two measures across the 12 texts in the MECO L1 database in the 13 different languages. Unsurprisingly, we observed a positive correlation between the two metrics; this is sensible as both are meant to provide measures of a word’s informativeness. However, these correlations were far from perfect (in the 0.5 to 0.65 range). We see this finding as an additional validation of our measure, while showing that it is still different than the one proposed by Fan and Reilly. For interested readers, we make available MECO texts with estimates by both operationalizations in the project’s OSF page.

**Figure S1**: The Relationship Between the Novel Informativeness Measure and Fan and Reilly's Metric, in 13 Languages. du: Dutch; ee: Estonian; en: English; fi: Finnish; ge: German; gr: Greek; he: Hebrew; it: Italian; ko: Korean; no: Norwegian; ru: Russian; sp: Spanish; tr: Turkish.

**
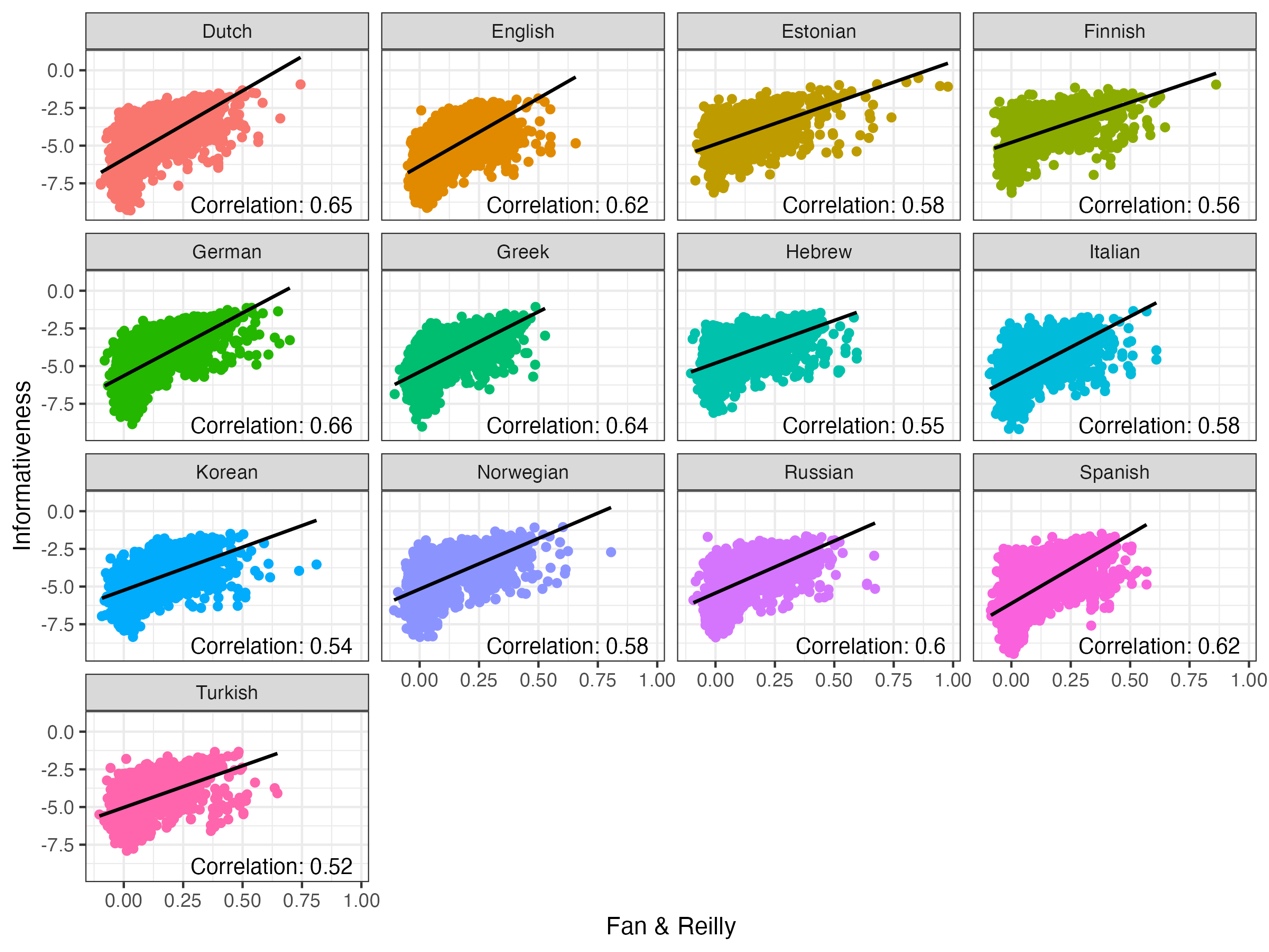
**

**Supplementary Materials S2: Effects of Informativeness on Early Eye-Movement Measures in the MECO-L2 Data**

In this section we report full outputs from models fitted to the MECO-L2 data for the early eye-movement measures – first run skipping, first fixation duration, and gaze duration.

***Dependent Variable: First Run Skipping***

In contrast to the results observed in L1 reading, informativeness did not have a significant effect on first run skipping (Table S2.1; $\beta= -0.0001, p= .99$; given the lack of a main effect for informativeness, we refrain from further interpretating interactions).

**Table S2.1:** Effects on Skipping, in the MECO L2 Data.

| Predictor | β | se | Chisq | DF | p |
| --- | --- | --- | --- | --- | --- |
| Word Length | -0.61 | 0.03 | 468.66 | 1 | $<.001$ |
| Log-transformed Frequency | 0.10 | 0.03 | 11.80 | 1 | $<.001$ |
| Predictability | -0.03 | 0.005 | 31.53 | 1 | $<.001$ |
| Surprisal | -0.006 | 0.006 | 1.02 | 1 | $.311$ |
| Informativeness | -0.0001 | 0.006 | 0.000 | 1 | $.99$ |
| Site |  |  | 195.39 | 11 | $<.001$ |
| Composite score | 0.08 | 0.03 | 9.76 | 1 | $.002$ |
| Composite score x Word Length | -0.05 | 0.005 | 88.89 | 1 | $<.001$ |
| Composite score x Log-transformed Frequency | -0.003 | 0.005 | 0.30 | 1 | $0.58$ |
| Composite score x Predictability | 0.02 | 0.003 | 42.87 | 1 | $<.001$ |
| Composite score x Surprisal | -0.007 | 0.004 | 3.22 | 1 | $.07$ |
| Composite score x Informativeness | 0.03 | 0.004 | 56.68 | 1 | $<.001$ |

***Dependent Variable: First Fixation Duration***

In the L2 analysis, although informativeness had a significant effect on first fixation duration (*p* = .01, Table S2.2), it was in the *opposite* direction than expected, with more informative words having somewhat *shorter* first fixation durations (a negative coefficient of $\beta= -0.003$). Given the lack of theoretical interpretability of this effect, its small magnitude, and the lack of a parallel effect in the L1 data, we presume that it reflects a Type-I error and/or is the outcome of multicollinearity in the model.

**Table S2.2:** Effects on First Fixation Duration, in the MECO L2 Data.

| Predictor | β | se | Chisq | DF | p |
| --- | --- | --- | --- | --- | --- |
| Word Length | 0.009 | 0.004 | 5.04 | 1 | $.025$ |
| Log-transformed Frequency | -0.009 | 0.004 | 4.85 | 1 | $.027$ |
| Predictability | -0.00002 | 0.0009 | 0.0004 | 1 | $.98$ |
| Surprisal | 0.004 | 0.007 | 13.84 | 1 | $<.001$ |
| Informativeness | -0.003 | 0.001 | 6.62 | 1 | $.01$ |
| Site |  |  | 23.41 | 11 | $.015$ |
| Composite score | -0.04 | 0.005 | 57.50 | 1 | $<.001$ |
| Composite score x Word Length | -0.002 | 0.0007 | 4.94 | 1 | $.026$ |
| Composite score x Log-transformed Frequency | -0.0003 | 0.0007 | 0.11 | 1 | $.74$ |
| Composite score x Predictability | -0.0006 | 0.0006 | 0.89 | 1 | $.34$ |
| Composite score x Surprisal | 0.0008 | 0.0006 | 1.72 | 1 | $.19$ |
| Composite score x Informativeness | -0.0009 | 0.0006 | 2.11 | 1 | $.15$ |

***Dependent Variable: Gaze Duration***

Again, in contrast to the MECO L1 analysis, informativeness did not have a significant effect on gaze duration (Table S2.3; $\beta=0.002, p= .06$).

**Table S2.3:** Effects on Gaze Duration, in the MECO L2 Data.

| Predictor | β | se | Chisq | DF | p |
| --- | --- | --- | --- | --- | --- |
| Word Length | 0.11 | 0.005 | 457.00 | 1 | $<.001$ |
| Log-transformed Frequency | -0.04 | 0.005 | 46.29 | 1 | $<.001$ |
| Predictability | -0.007 | 0.001 | 9.14 | 1 | $.002$ |
| Surprisal | 0.18 | 0.007 | 219.39 | 1 | $<.001$ |
| Informativeness | 0.002 | 0.002 | 3.44 | 1 | $.06$ |
| Site |  |  | 45.99 | 11 | $<.001$ |
| Composite score | -0.07 | 0.007 | 109.80 | 1 | $<.001$ |
| Composite score x Word Length | -0.02 | 0.0008 | 860.62 | 1 | $<.001$ |
| Composite score x Log-transformed Frequency | 0.003 | 0.0009 | 9.51 | 1 | $.002$ |
| Composite score x Predictability | 0.002 | 0.0007 | 4.97 | 1 | $.025$ |
| Composite score x Surprisal | 0.01 | 0.004 | 3.32 | 1 | $.068$ |
| Composite score x Informativeness | -0.001 | 0.0008 | 3.14 | 1 | $.076$ |

**References**

Fan, X., & Reilly, R. (2020). Reading development at the text level: an investigation of surprisal and embedding based text similarity effects on eye movements in Chinese early readers. *Journal of Eye Movement Research*, *13*(6).

Reimers, N., & Gurevych, I. (2019). Sentence-bert: Sentence embeddings using siamese bert-networks. *arXiv preprint arXiv:1908.10084*.

Reimers, N., & Gurevych, I. (2020). Making monolingual sentence embeddings multilingual using knowledge distillation. *arXiv preprint arXiv:2004.09813*.
